# Roots applicable, high sensitivity and specificity assay for the detection of *Candidatus* Liberibacter asiaticus in *citrus* roots and fruits

**DOI:** 10.5511/plantbiotechnology.23.1129a

**Published:** 2024-03-25

**Authors:** Zecheng Zhong, Yu Chen, Jinhua Liu, Wei Wang, Feng Zhou, Liu Hu, Jinlian Zhang, Tingsu Chen, Jiyu Xiang, Tingdong Li, Yingbin Wang, Shiyin Zhang, Shengxiang Ge, Jun Zhang, Ningshao Xia

**Affiliations:** 1 National Institute of Diagnostics and Vaccine Development in Infectious Diseases, School of Public Health, Xiamen University, Xiamen 361102, China; 2 School of Medicine, Xiamen University, Xiamen 361102, China; 3 Zhejiang Yang Sheng Tang Institute of Natural Medicine Co., Ltd., Hangzhou 310024, China; 4 Microbiology Research Institute, Guangxi Academy of Agricultural Sciences, Nanning 530007, China

**Keywords:** ‘*Candidatus* Liberibacter asiaticus’, early diagnosis, HLB, 16S rRNA

## Abstract

*Candidatus* Liberibacter asiaticus (CLas), a phloem-limited Gram-negative bacterium, is associated with citrus huanglongbing (HLB), which is one of the most destructive diseases currently threatening citrus production worldwide. No effective treatment for HLB is currently available. Effective prevention and control in the initial stage can block the spread and disease progression of HLB. Herein, we developed a co-detection assay for the 16S rDNA and 16S rRNA of CLas, the sensitivity of the co-detection assay was significantly increased over that of the single CLas DNA detection system. Beyond this, we found that the co-detection assay was a better fit to the root samples with higher population abundance than the previous reported detection system because it has a better specificity. Moreover, we found that the contents of 16S rRNA of CLas in citrus roots and fruits are significantly higher than that in leaves, which suggests that the time of HLB diagnosis is probably earlier by using these special tissues and the replication of CLas may become more active in these tissues, further suggested that the significance of study the mechanism of infection, prevention and control of HLB staring from these tissues.

## Introduction

‘*Candidatus* Liberibacter asiaticus’ (CLas) is a phloem-limited Gram-negative bacterium that is associated with yellow shoot disease, also known as huanglongbing (HLB) (Bové 2006). After infection with CLas, symptomatic fruit are small, light, more acidic, with decreased percentage in juice content (Baldwin et al. 2010; Bassanezi et al. 2009). With the increased agricultural globalization, citrus HLB has spread to Asia, Africa, the United States, and other citrus production areas, becoming the most destructive citrus pathogen threatening the global citrus industry (Gottwald 2010; Gottwald et al. 2007).

HLB is now considered to be associated with three uncultivable Gram-negative bacteria: *Ca*. L. asiaticus, *Ca*. L. africanus, and *Ca*. L. americanus (Duan et al. 2009; Jagoueix et al. 1994; Teixeira et al. 2005). To date, there is still no specific treatment to inhibit CLas proliferation in citrus or completely eliminate these bacteria. Currently, in order to alleviate the loss of citrus production caused by CLas infection, plantations are currently managed via the removal of infected trees, insect control (e.g., the citrus psyllid), and sterilization of scions (Zheng et al. 2018). Effective prevention and control of CLas infection and transmission is one of the best ways to remove and control HLB. Nevertheless, the success of these measures depend heavily on high sensitive diagnostic techniques. Detection approaches, such as symptomatology (Gómez 2008; Wulff et al. 2019), biological indexing (Razi et al. 2012), iodine reaction technique (Schneider 1968; Takushi et al. 2007), light or electron microscopy (Mishra et al. 2012; Secor et al. 2009), and immunology (Ding et al. 2016), have been used to diagnose HLB; however, these methods are not universal for low sensitivity or specificity.

The widely accepted method used for the identification of CLas is polymerase chain reaction (PCR) based assays (Aksenov et al. 2014; Bastianel et al. 2005). As technology advances, real-time fluorescence quantitative PCR with high sensitivity and specificity plays an important role in the diagnosis of HLB (Li et al. 2006; Manjunath et al. 2008; Wang et al. 2006). Interestingly, we noticed that the DNA of CLas was the primary target for detection. However, previous research has demonstrated that using the 16S rRNA of CLas as a target can enhance sensitivity (Kim and Wang 2009). Nevertheless, there is a lack of systematic research on suitable plant tissues for the early diagnosis of CLas using 16S rRNA. Additionally, relying solely on RNA detection poses inherent risks due to the known instability of RNA (Stevenson et al. 2015). In cases where RNA degradation occurs, the effectiveness of the assay sensitivity may be compromised compared to DNA detection. Therefore, a more optimal approach would be the simultaneous detection of both 16S rDNA and 16S rRNA of CLas in the actual application process. For example, when detecting only 16S rRNA, which may be prone to degradation and result in decreased sensitivity, the co-detection method can mitigate the decrease in CLas detection sensitivity. On the other hand, when 16S rRNA degradation is not a concern, the 16S rDNA and 16S rRNA co-detection method can fully leverage the advantage of high RNA abundance to improve CLas detection sensitivity. However, there have been no related studies in this area. Moreover, previous studies were not adequately explored the value of RNA detection. Apart from enhancing detection sensitivity, RNA detection can also serve as an indicator of CLas transcriptional activity, providing valuable insights into the life activities and pathogenic mechanisms of CLas. Nonetheless, the existing RNA detection techniques have been limited to leaf detected, impeding researchers from investigating related mechanisms comprehensively.

To address the above question, we have established a novel, highly sensitivity, and highly specificity co-detection method for CLas 16S rDNA and 16S rRNA. This method is applicable for CLas detection in various scenarios and has the potential to be an important tool for early diagnosis of CLas. Additionally, this method is widely applicable for different tissues of citrus (include root, fruit, leaf and stem). Within these, we further analyzed differential 16S rRNA expression quantity in the comparison of the different tissues of the same diseased plants to obtain the site of active replication of CLas, thereby providing a basis for efficiently governance of HLB.

## Materials and methods

### Primer design

Taking into consideration that the copy number of the 16S rDNA gene sequence is three times higher than other genes (Duan et al. 2009), primers and probes designed targeting the 16S rDNA gene have been widely utilized (Li et al. 2006). Therefore, we have chosen the 16S rDNA gene as the target. The 16S rDNA sequences of CLas and its close relatives and other unrelated species genes were downloaded from National Center for Biotechnology Information (NCBI) GenBank (https://www.ncbi.nlm.nih.gov). The ClustalW program in MEGA 5.0 was used to align these sequences and the alignment was used for primers/probe design (Tamura et al. 2011). Newly designed PCR primers/probe (Supplementary Figure S2) targeting highly conserved regions were designed using the Oligo 6 software. The specificity of the primers was checked using the Primer-Blast tool (http://www.ncbi.nlm.nih.gov/tools/primer-blast/).

### Plant material

The tissues (including leaves, stems, roots, and fruits) of CLas-infected Navel orange trees (approximately 5 years old) were collected from Ganzhou, Jiangxi, China. The healthy leaves and roots samples were collected from Navel orange trees, Orah mandarin, Lemon tree and citrus poonensis cultured in greenhouse from different areas. The samples were collected in plastic bags with respiration holes and sent to the laboratory within 24 hours. The leaves and roots of other *Rutaceae* and non-*rutaceae* species were collected from a suburb near Xiamen University, Fujian, China, where Navel orange trees have not been planted on a large scale.

### Nucleic acid extraction

Three leaves were randomly selected from a Navel orange tree and the midribs and petioles were chopped into small pieces with a small pair of scissors. 150 mg of leaf pieces was collected into a 2 ml eppendorf (EP) tube. Three stems selected from a Navel orange tree were also preprocessed in this way (150 mg of pieces). For roots, the new apical tissue was randomly selected and cut into fragments (150 mg of pieces). For fruit, the core tissues of the fruit was prepared for nucleic acid extraction (150 mg of pieces). To prevent cross-contamination, a separate pair of scissors for each sample was used. Later, a steel ball (Solarbio, Beijing, China) with a diameter of 5 mm was added to each EP tube and all of the EP tubes were simultaneously collected in a foam box. Then the foam box was mixed upside down after the liquid nitrogen was added and the fragments were ground into powder. Then, 700 µl of cetyl trimethyl ammonium bromide (CTAB, AMRESCO, WA, USA) extraction buffer, 10 µl proteinase K (GenMagBio, Beijing, China), and 10 µl dithiothreitol (DTT, Xilong Scientific, Shanghai, China) were added, mixed by inversion, and incubated at 65°C in a heating block (with shaking twice) for 30 min. The sample was then removed from the heating block and spun at 12,900 rpm for 1 min. 600 µl of supernatant was transferred into a new 2 ml EP tube, isochoric phenol : chloroform : isopentanol (25 : 24 : 1, Xilong Scientific) was added, and the sample was mix by inversion to form emulsion. It was then spun at 12,900 rpm for 3 min, following which 500 µl of supernatant was added into a new 2 ml EP tube to which isochoric chloroform : isopentanol (24 : 1, Xilong Scientific) was pre-added. The sample was then mixed by inversion to form an emulsion and spun at 12,900 rpm for 2 min. 400 µl of supernatant was transferred into a new 1.5 ml EP tube, isochoric isopropyl alcohol (Xilong Scientific) and 1/10 volume of sodium acetate (Xilong Scientific) was added, and it was incubated at −20°C for 10 min. After removal from the freezer, the sample was mixed by inversion and spun at 12,000 rpm for 10 min. It was then drained and washed with 75% ethanol (Xilong Scientific), spun at 12,000 rpm for 2 min, and the ethanol was removed. Tubes were dried to remove the remaining ethanol and the total nucleic acid was suspended in 50 µl elution buffer (10 mM Tris-HCl and 1 mM EDTA) and immediately stored at −20°C for later use.

### Standard RNA preparation

The plasmid with the 16S rRNA genes contained a T7 promoter sequence were synthesized by a manufacturer (Sangon, Shanghai, China). Single-strand RNA was transcribed using Thermo in vitro transcription kit (Thermo Fisher Scientific, Waltham, MA, USA). After that, RNase-free DNase I (TaKaRa, Tokyo, Japan) was used for DNase treatment of transcription products. The Multiskan Spectrum spectrophotometer (Thermo Fisher Scientific) was used to determine the concentrations and the quality of the RNA, the RNA was storage at −20°C before use.

### PCR amplification

For single DNA detection assay, the self-designed primers/probe (YSD-F/YSD-P/YSD-R) and previously reported primers/probe (Supplementary Table S1) were used to perform qPCR analysis using CFX96 Real-Time system (Bio-Rad Laboratories, Hercules, CA, USA). The 5′ end of the probe labeled with 6-FAM fluorophore and the 3′ end of the probe labeled with BHQ_1_. The reaction system was conducted in a total volume of 25 µl, containing 5 µl of extracted nucleic acid template, 2.5 µl of 10× buffer (TaKaRa), a mixture of 250 mmol l^−1^ of dNTP (TaKaRa), 400 µmol ml^−1^ of primers and probe (Sangon), 1 U of Taq HS DNA polymerase (TaKaRa). The reaction was initiated by heating to 95°C for 5 min, followed by 45 cycles of 95°C for 15 s, and 55°C for 50 s. Fluorescence was recorded at the annealing step during the 45 cycles.

For co-detection assay, primers were the same as in the single DNA detection assay but additional 16 U TransScript Reverse Transcriptase (TransGen Biotech, Beijing, China) was added into the 25 µl reaction system. A reverse transcription process of 10 min at 45°C was required before the amplification procedure.

### CLas 16S rRNA quantification in different tissues of citrus trees

In order to quantitative CLas 16S rRNA in different tissues of citrus trees, the leaves, stems, roots, and fruits were collected from CLas positive citrus trees, and the nucleic acid (include DNA and RNA) was extracted by CTAB method. DNA digestion was performed using the RNase-free DNase I (TaKaRa), after purification, the RNA template was used to perform quantification detection by co-detection assay. When quantification of 16S rDNA, the other equal part template (No DNase I digested) was diluted to a same grade and detected by single DNA detection assay.

## Results

### Designed new primers and probe for 16S rRNA of CLas

Previous literature reported that the widely used primers and probe targeting 16S rRNA gene of CLas has a high sensitivity (Cellier et al. 2020; Li et al. 2006). These researches essentially selected leaves as the experimental subject. Interestingly, we found that this primers and probe were not suitable for root samples detection. NCBI BLASTn results showed that the forward primer and probe of the previous work (Li et al. 2006) matched with the genes of other not relevant organisms besides CLas (Supplementary Figure S1A, B). Furthermore, we have used the primer and probe mentioned above to detect the leaves and roots of 10 healthy Navel orange trees, results showed that 1 of 10 leaves samples and 2 of 10 roots samples were positively detected (Supplementary Table S2). Given this, we need to redesign new and better specificity primers and probe for 16S rRNA gene of CLas, to meet the immediate needs of high sensitivity and specificity detection for CLas.

The results of multiple sequence alignment showed that the newly designed primers were more complementary pairing to CLas than the *Ca*. L. africanus, *Ca*. L. americanus, *Ca*. L. solanacearum and other closely related strains and unrelated species genes (Supplementary Figure S2). To fully verify the specificity of our newly designed primer and avoid cross-reactions, we did a BLASTn search in NCBI and found that the primer-probe has a 100% sequence coverage with 100% identity to the CLas of 16S rDNA (Supplementary Figure S1C, D, E).

### Sensitivity and linearity of co-detection assay for CLas

**Figure 1:**
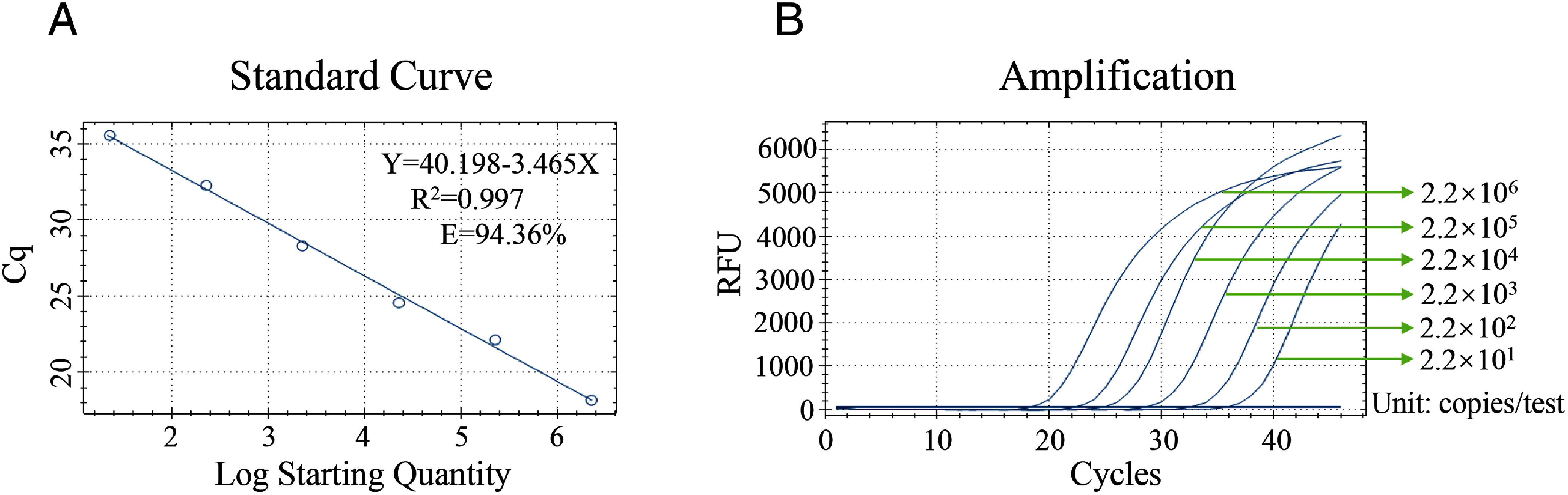
Figure 1. Sensitivity and linearity of co-detection assay for CLas. A A standard curve showing the relationship between the starting template concentration and Cq values. B The sensitivity of the co-detection assay, 10-fold serial dilution of the mixture of plasmid DNA and in vitro transcribed RNA containing the target sequence was used.

After further optimization, we preliminarily set up the co-detection assay. Immediately after, a set of 10-fold gradient dilutions of the in vitro transcribed RNA was used as template to evaluate the linearity of co-detection assays. The R^2^ value of the standard curve line fit by the regression analysis was 0.997, and the PCR efficiency for this assay was 94.36% according to the formula E=[10^−1/slope^] −1 (Figure 1A). The lowest detection limit of the 10-fold gradient dilution of RNA was used as the starting point and the sensitivity limit of the assay was 22 copies/test (Figure 1B). In addition, several experiments (*n*=20) with a 2-fold dilution series of template was utilized. For the gradient of the endpoint dilution, the concentration at which a positive amplification signal issued by at least 19 replicates (95%). We found that only 15 replicates showed positive amplification signal when the RNA concentration was 11 copies/test. Lastly, the 95% lower limit of the co-detection assay was defined as 22 copies/test.

### Comparing the lower limit of co-detection assay with single DNA detection assays

**Figure 2:**
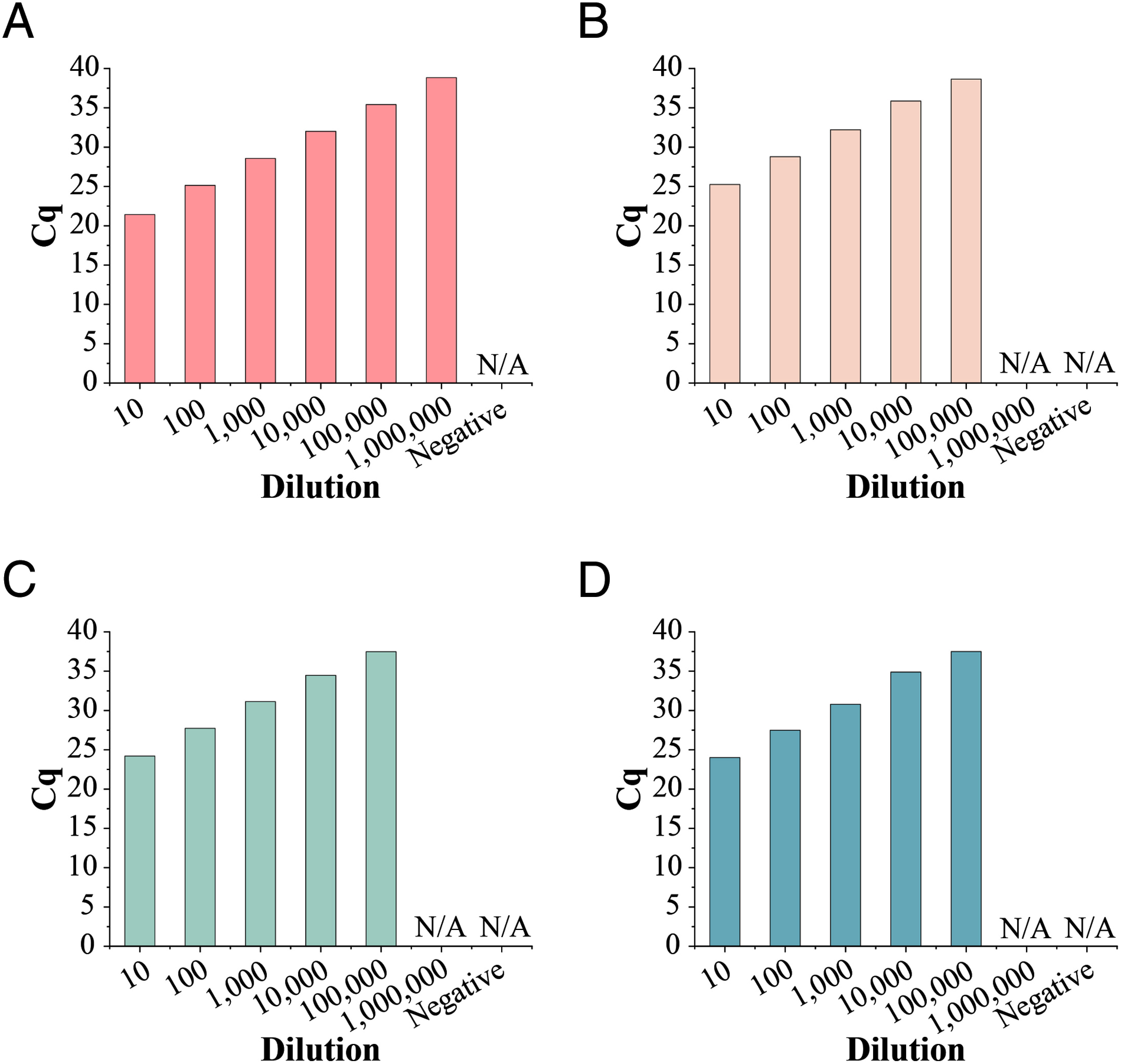
Figure 2. Comparison the lower limit of detection of co-detection assay and single DNA detection assays. Ten-fold dilution series of template were detected by four different assays, respectively. A Co-detection assay with YSD-FRP. B Single DNA detection assay with YSD-FRP. C Single DNA detection assay with HLBasfP. D Single DNA detection assay to detect nrdB gene. N/A means not detected. *n*=3 reactions per gradient.

Next, we compared the lower limit of co-detection assay with single DNA detection assays. The earlier reported real-time PCR primer targeting 16S rDNA of CLas (Li et al. 2006) and targeting *nrdB* of CLas (Zheng et al. 2016) were selected to evaluation. The nucleic acid template was extracted from the leaves of CLas infected Navel orange trees, 10-fold gradient dilution template was detected by four different assays, respectively. Results showed that the sensitivity of our co-detection assay was 10 times higher than single DNA detection assays (Figure 2A–D). Results suggests that the 16S rRNA content in these samples is approximately 10 times higher than that of 16S rDNA, which in turn implies that the detection performance of our RNA assay is approximately 10 times higher than that of DNA assay.

### Evaluation of co-detection assay specificity

To verify the specificity of our new co-detection system sufficiently, the roots and leaves of 10 healthy independent Navel orange trees and other healthy non-rutaceae species were collected. The total nucleic acid (including DNA and RNA) was extracted and detected by newly designed YSD-FRP and previously reported HLBasfP qRT-PCR assay (Li et al. 2006). The results showed that no unspecific signal was detected in the roots and leaves of 10 healthy Navel orange trees, roots of 15 healthy non-rutaceae plants and leaves of 40 healthy non-rutaceae plants by using the YSD-FRP qRT-PCR assay (Supplementary Table S2). However, when we detected the roots of 10 healthy Navel orange trees by previous reported HLBasfP qRT-PCR assay, we found that 2 roots samples and 1 leaf sample were false-positive, besides, positive fluorescent signals were detected in the leaves of *Polygonum L* and *Cogongrass*. The BLAST alignment results of amplicon sequences confirmed that these 5 nonspecific amplification products were matched for non-CLas (Supplementary Figure S3).

Furthermore, we collected the roots of healthy Navel orange trees, healthy Orah mandarin trees, healthy Lemon tree trees and healthy citrus poonensis trees from different areas to further evaluate the specificity of the YSD-FRP assay. The results showed that both co-detection assay and single DNA detection assay of YSD-FRP do not rise to any unspecific signal when compared with HLBasfP assays (Supplementary Figure S4, Table S3). All of these results suggested that the specificity of YSD-FRP assays are better than that of HLBasafpr assays and more applicable for roots detection.

### Co-detection assay could improve the positive detection rates of CLas

**Figure 3:**
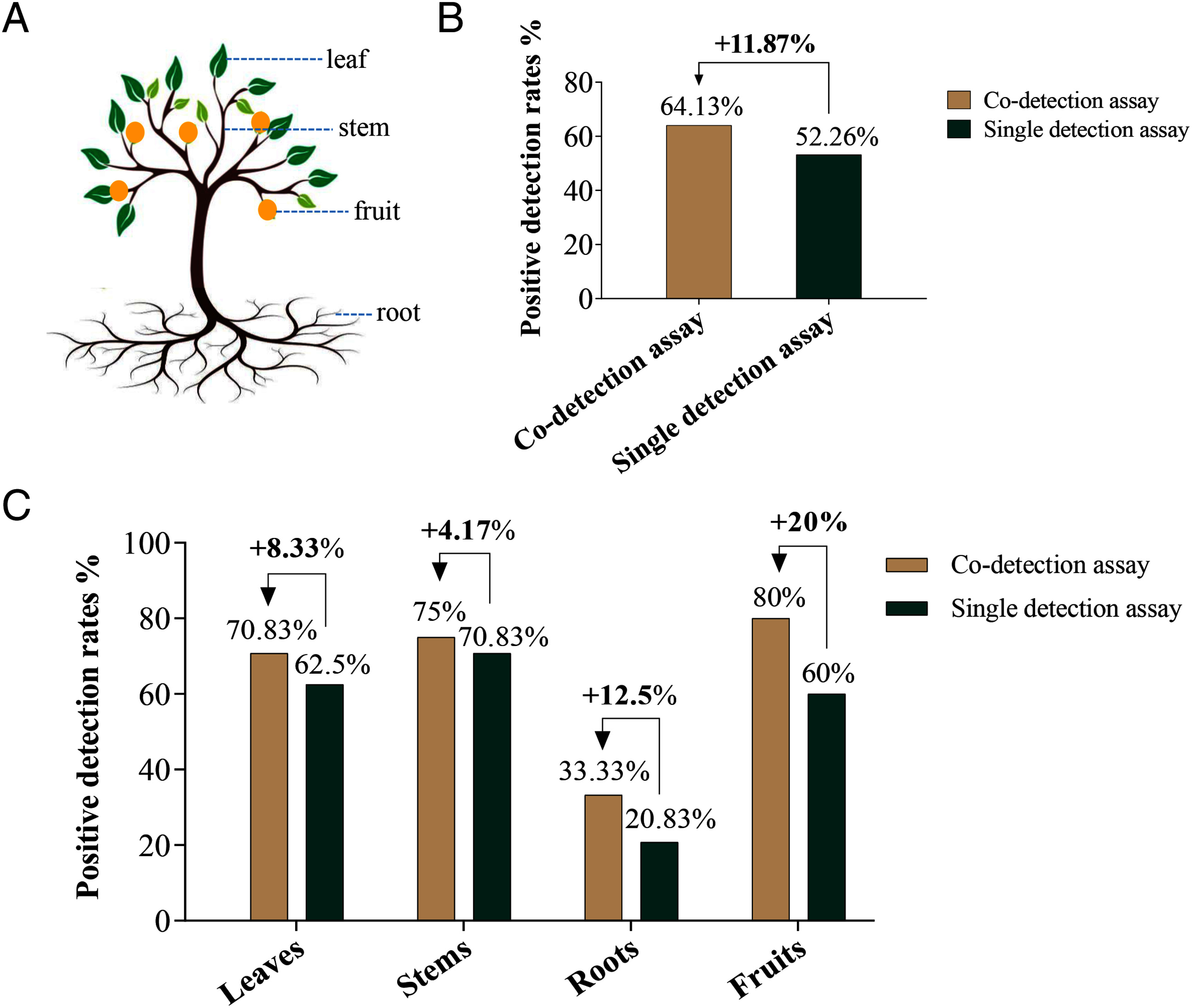
Figure 3. Comparison of positive detection rates between co-detection assay and the single detection assay. A Different tissues included leaf, stem, fruit, and root were collected from CLas infected Navel orange trees. B The overall comparison of positive detection rates between the co-detection assay and the single DNA detection assay, and C the comparison of the detection rate of CLas in different tissues of citrus trees were shown.

Next, we evaluated the positive detection rates of CLas at large sample size. 92 samples, included the 24 leaves, 24 stems, 24 roots, and 20 fruits, were collected from 6 CLas infected Navel orange trees (Figure 3A). Nucleic acid (including DNA and RNA) was extracted and tested by co-detection assay and single DNA detection assay of YSD-FRP. The results showed that the positive detection rate of CLas tested by co-detection assay was 11.87% higher than that detected with single DNA detection assay (Figure 3B). Intriguingly, we noted that no matter whether the samples were leaves, stems, roots, or fruits, the positive detection rate of CLas detected by co-detection assay was always higher than single DNA detection assay. Specifically, 8.33% higher for leaves, 4.17% higher for stems, 12.5% higher for roots, 20% higher for fruits (Figure 3C).

### Comparing the expression of 16S rRNA of CLas in leaves, stems, roots, and fruits

**Figure 4:**
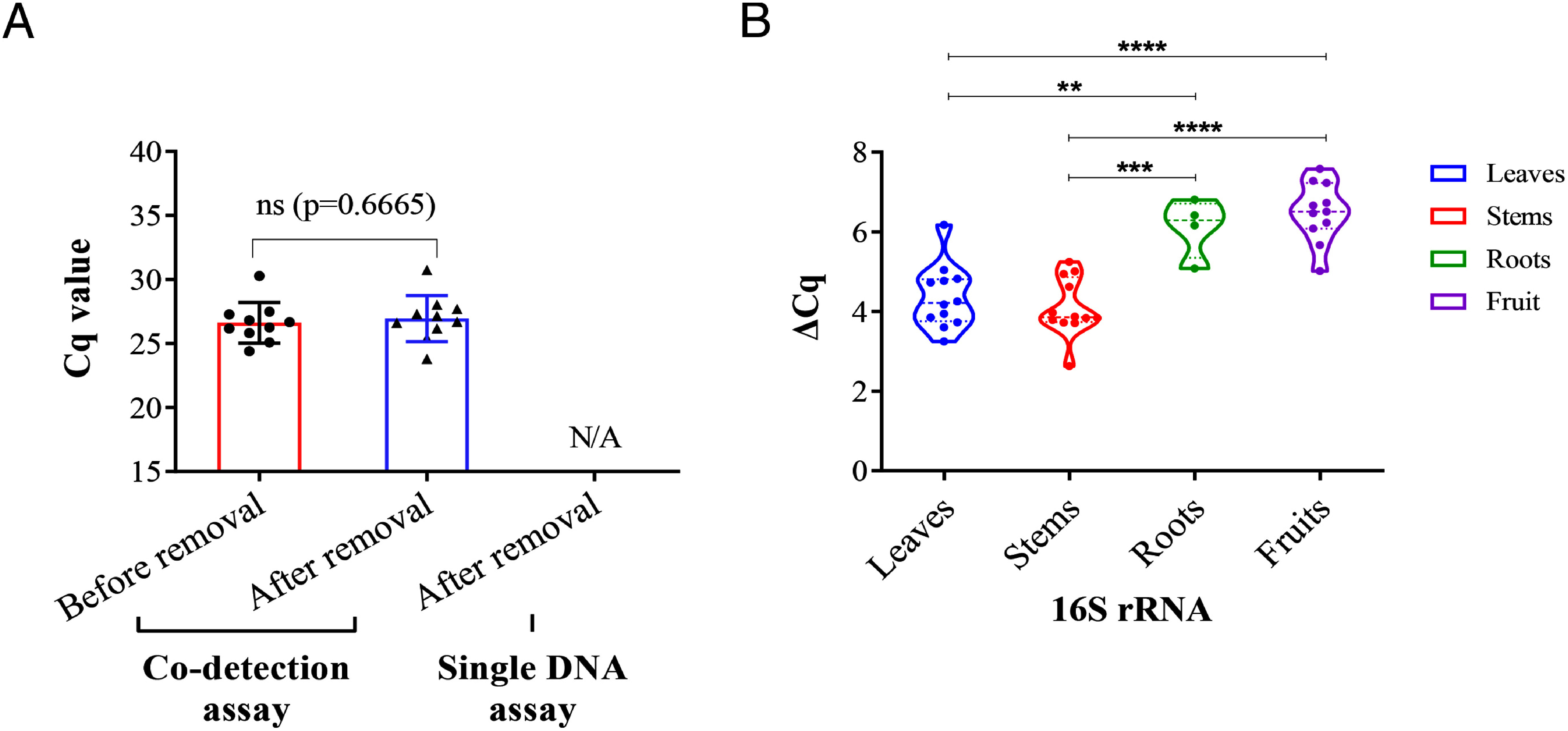
Figure 4. The differences in the content of CLas 16S rRNA in different tissues of citrus. A Results confirmed that DNase I was able to remove DNA from template pools without causing RNA degradation. B After removed DNA, the 16S rRNA was detected by using co-detection assay of YSD-FRP. The template extracted by CTAB method was diluted to the same grade and detected by single DNA detection assay of YSD-FRP. ΔCq stand for the Cq value of the single DNA detection assay minus the Cq value of the co-detection assay. “N/A” means undetectable. The “ns” means not statistically significantly different. Asterisks indicate statistically significant differences (∗∗*p*<0.01, ∗∗∗*p*<0.001, ∗∗∗∗*p*<0.0001).

To compare the expression of 16S rRNA of CLas in leaves, stems, roots, and fruits, we randomly selected 12 CLas infected Navel orange trees, the leaves, stems, roots, and fruits of these trees were collected, respectively. The nucleic acid was extracted and divided into two equal portions. When performed 16S rRNA detection, one portion of nucleic acids was treated with DNase I first to remove 16S rDNA contamination and then detected by co-detection assay of YSD-FRP. When performed 16S rDNA detection, another portion of nucleic acid was diluted to the same level and then detected by single-detection assay of YSD-FRP. Although RNA is less stable and more susceptible to degradation compared to DNA (Stevenson et al. 2015), the results showed that 16S rDNA was successfully removed while not affect the 16S rRNA detection (Figure 4A). This result also suggests that we have protected 16S rRNA well the time between sample collection and detection. Furthermore, we found that the difference of ΔCq between leaves and roots, leaves and fruits, stems and roots, or stems and fruits were significant (Figure 4B), which meaning that the 16S rRNA content of CLas in the roots and fruits was greater than that in the other tissues.

## Discussion

At present, there is no specific treatment that kills CLas, causing the pathogen to remain in citrus trees for a long time, which influences the quality of citrus trees as well as reducing the yield of oranges. Moreover, the infected trees will become a long-term source of infection. Early detection of CLas and cut down diseased trees can effectively eliminate the infectious sources and reduce financial losses, while it depends heavily on high-sensitive determination. Quantitative PCR (qPCR) technology, quickly and accurately quantify CLas, has shown promise to achieve early diagnostics in roots sample detection (Johnson et al. 2014; Park et al. 2018). However, we found previously reported high sensitivity CLas 16S rRNA detection assay was not apply to roots sample detection due to the non-specific amplifications (Supplementary Figure S3, Tables S2, S3, S4), which could be interpreted as the similarity of 16S rRNA gene sequence among different CLas related strains caused unspecific binding.

A new pair of primers and a probe based on the 16S rRNA gene of CLas was redesign. NCBI BLASTn results showed that the nucleotide sequences of primers and a probe were only matched with CLas (Supplementary Figure S1C, D, E). Moreover, the specificity of the primers and probe were further verified by 10 healthy Navel orange roots and 15 healthy non-rutaceae plants roots and 27 healthy citrus plants roots, the results showed that no false-positive signal was generated (Supplementary Figure S4, Tables S2, S3). These results well illustrated that the excellent specificity of the new designed primers and probe. On the other hand, the lower limit of the co-detection assay was an order of magnitude higher than single DNA detection assay (Figure 2), not only that, the positive rate of CLas in co-detection assay was higher than single DNA detection assay for real samples detection has also been confirmed (Figure 3B). All of these results demonstrated that the co-detection assay is more suitable for roots sample detection because of the better performances in sensitivity and specificity. In fact, there have been literature reports that CLas preferentially colonize the roots (Thapa et al. 2021), and the detection of the CLas occurred earlier, more consistently, and more frequently in root samples than in leaf samples (Braswell et al. 2020). From this perspective, our co-detection method can provide an effective tool in root samples detection.

Co-detection assay was able to improve the positive rate of CLas compared with single DNA detection assay (Figure 3B), interestingly, the percentage improvement in roots and fruits were greater than leaves and stems (Figure 3C). These results could be explained by the higher expression of the 16S rRNA of CLas in roots and fruits (Figure 4B). Further, the higher expression of the 16S rRNA of CLas in these tissues which may be connected with the fact that CLas shows a preference for reproduction in these tissues environment, indicating a better living environment for the pathogen. Previous complete genome sequence results showed that CLas lack genes to produce certain amino acids, which must be obtained from the host cells (Duan et al. 2009; Wang and Trivedi 2013); root and fruit may provide a better niche for growth and for active proliferation. Root play an important role in plant growth and survival by providing water, CO_2_, inorganic matter, N_2_, etc. (Barberon et al. 2016). While these functions are inseparable from the contribution of microorganisms, the diversity and stability of unseen bacterial communities present in the rhizosphere heavily influence plant quality (van der Heijden et al. 2008). Changes in plant physiology mediated by CLas infection could elicit drastic shifts in the composition and functional potential of soil microbial communities (Trivedi et al. 2012), thus causing a series of changes in plant nutrition level and growth state. Physiological changes mediated by CLas might have important implications for the productivity and sustainability of citrus.

Comprehending the pathological and physiological characteristics of CLas can bring a better understanding of the interactions between CLas and its citrus host. However, this researches highly relied on in vitro culturing of CLas, which is still not applicable currently. Some effort has been made to achieve this goal, for example, Liber A medium. As reported, Liber A medium is the most successful in vitro culturing medium for CLas right so far, but this technique still has many limitations. Liber A medium was made by series of complex and time-consuming operations which start from extracting nutrients from petioles and mid of veins of young citrus leaves (Sechler et al. 2009). Although it is very difficult to repeat Liber A medium, and it is only a nutrient-supporting medium which only support the living of CLas in two weeks, Liber A medium proves that CLas required extra nutrients or chemical signal from host to support its efficiently growth in vitro. Jennifer K proved that juice-added culturing medium can prolong the viability of CLas for several months (Parker et al. 2014), Marcus V used wide range of compounds to improve juice and incubated it under different conditions to optimize the growth of CLas (Merfa et al. 2022). These studies indicate CLas may have nutritional or environmental preference for this medium over another. In this study, a high expression of CLas 16S rRNA in citrus fruits is consistent with the preference of culturing medium. But chemical signals which was required for CLas culturing and could be found in the host may be missing in culture mediums, resulting in the failure of a truly significant in vitro culture (Merfa et al. 2019). Exploring key factors for high expression of CLas 16S rRNA in roots and fruits may help the development in long term in vitro culturing for CLas. Meanwhile, our co-detection system can provide a good detection method for the screening of key chemical signals.

In summary, we constructed a highly sensitive co-detection assay that could improve the positive detection rate of CLas. Besides, we discovered that the contents of 16S rRNA of CLas in roots and fruit were significantly higher than that in leaves. This finding may have great significance in the following two aspects. First, given that RNA transcription is associated with the energetic states of the bacterium, the high expression of RNA of CLas in roots suggested that the root of plants might be an active site for the replication of CLas, further suggested that the significance of study the mechanism of infection and HLB prevention and control staring with root. Second, the high expression of RNA of CLas in roots also suggests that the time of HLB diagnosis is probably earlier by using root samples. There have been researches report on the importance of roots as materials for early diagnosis of CLas (Braswell et al. 2020; Thapa et al. 2021). Importantly, the higher sensitivity co-detection assay is more suitable for root samples might play a more important role in early diagnosis of HLB. This is of great significance in the primary prevention and control of CLas.

## Abbreviations

CLas: *Candidatus* Liberibacter asiaticus
CTAB: cetyl trimethyl ammonium bromide
DTT: dithiothreitol
EP: eppendorf
HLB: huanglongbing
NCBI: National Center for Biotechnology Information
PCR: polymerase chain reaction
qPCR: quantitative PCR

## References

[RAksenov2014] Aksenov AA, Pasamontes A, Peirano DJ, Zhao W, Dandekar AM, Fiehn O, Ehsani R, Davis CE (2014) Detection of huanglongbing disease using differential mobility spectrometry. *Anal Chem* 86: 2481–2488 24484549 10.1021/ac403469y

[RBaldwin2010] Baldwin E, Plotto A, Manthey J, McCollum G, Bai JH, Irey M, Cameron R, Luzio G (2010) Effect of Liberibacter infection (huanglongbing disease) of citrus on orange fruit physiology and fruit/fruit juice quality: Chemical and physical analyses. *J Agric Food Chem* 58: 1247–1262 20030384 10.1021/jf9031958

[RBarberon2016] Barberon M, Vermeer JE, De Bellis D, Wang P, Naseer S, Andersen TG, Humbel BM, Nawrath C, Takano J, Salt DE, et al. (2016) Adaptation of root function by nutrient-induced plasticity of endodermal differentiation. *Cell* 164: 447–459 26777403 10.1016/j.cell.2015.12.021

[RBassanezi2009] Bassanezi RB, Montesino LH, Stuchi ES (2009) Effects of huanglongbing on fruit quality of sweet orange cultivars in Brazil. *Eur J Plant Pathol* 125: 565–572 10.1094/PDIS-91-11-1407 30780746

[RBastianel2005] Bastianel C, Garnier-Semancik M, Renaudin J, Bové JM, Eveillard S (2005) Diversity of “*Candidatus* Liberibacter asiaticus,” based on the *omp* gene sequence. *Appl Environ Microbiol* 71: 6473–6478 16269671 10.1128/AEM.71.11.6473-6478.2005 PMC1287744

[d67e706] Bové JM (2006) Huanglongbing: A destructive, newly-emerging, century-old disease of citrus. *J Plant Pathol* 88: 7–37

[RBraswell2020] Braswell WE, Park JW, Stansly PA, Kostyk BC, Louzada ES, da Graca JV, Kunta M (2020) Root samples provide early and improved detection of *Candidatus* Liberibacter asiaticus in Citrus. *Sci Rep* 10: 16982 33046775 10.1038/s41598-020-74093-x PMC7550583

[RCellier2020] Cellier G, Redondo C, Cubero J, Roselló M, de Andrade E, Cruz L, Ince E, Yildiz HN, Güler PG, D’Onghia AM, et al. (2020) Comparison of the performance of the main real-time and conventional PCR detection tests for ‘*Candidatus* Liberibacter’ spp., plant pathogenic bacteria causing the huanglongbing disease in *Citrus* spp. *Eur J Plant Pathol* 157: 919–941

[RDing2016] Ding F, Duan Y, Yuan Q, Shao J, Hartung JS (2016) Serological detection of ‘*Candidatus* Liberibacter asiaticus’ in citrus, and identification by GeLC-MS/MS of a chaperone protein responding to cellular pathogens. *Sci Rep* 6: 29272 27381064 10.1038/srep29272 PMC4933950

[RDuan2009] Duan YP, Zhou LJ, Hall DG, Li WB, Doddapaneni H, Lin H, Liu L, Vahling CM, Gabriel DW, Williams KP, et al. (2009) Complete genome sequence of citrus huanglongbing bacterium, ‘*Candidatus* Liberibacter asiaticus’ obtained through metagenomics. *Mol Plant Microbe Interact* 22: 1011–1020 19589076 10.1094/MPMI-22-8-1011

[d67e809] Gómez HD (2008) *Experiencies on HLB (Huanglongbing) Symptoms Detection in Florida*. 1st International Workshop on Citrus Huanglongbing (Canditatus Liberibacter spp.) and Asian Citrus Psyllid (Diaphorina citri), Hermosillo, Sonora, México

[RGottwald2010] Gottwald TR (2010) Current epidemiological understanding of citrus huanglongbing. *Annu Rev Phytopathol* 48: 119–139 20415578 10.1146/annurev-phyto-073009-114418

[RGottwald2007] Gottwald TR, Graça JV, Bassanezi RB (2007) Citrus huanglongbing: The pathogen and its impact. *Plant Health Prog* 8: 31

[RJagoueix1994] Jagoueix S, Bove JM, Garnier M (1994) The phloem-limited bacterium of greening disease of citrus is a member of the alpha subdivision of the *Proteobacteria.* *Int J Syst Bacteriol* 44: 379–386 7520729 10.1099/00207713-44-3-379

[RJohnson2014] Johnson EG, Wu J, Bright DB, Graham JH (2014) Association of ‘*Candidatus* Liberibacter asiaticus’ root infection, but not phloem plugging with root loss on huanglongbing-affected trees prior to appearance of foliar symptoms. *Plant Pathol* 63: 290–298

[RKim2009] Kim JS, Wang N (2009) Characterization of copy numbers of 16S rDNA and 16S rRNA of *Candidatus* Liberibacter asiaticus and the implication in detection *in planta* using quantitative PCR. *BMC Res Notes* 2: 37 19284534 10.1186/1756-0500-2-37 PMC2663771

[RLi2006] Li W, Hartung JS, Levy L (2006) Quantitative real-time PCR for detection and identification of *Candidatus* Liberibacter species associated with citrus huanglongbing. *J Microbiol Methods* 66: 104–115 16414133 10.1016/j.mimet.2005.10.018

[RManjunath2007] Manjunath KL, Halbert SE, Ramadugu C, Webb S, Lee RF (2008) Detection of ‘*Candidatus* Liberibacter asiaticus’ in *Diaphorina citri* and its importance in the management of citrus huanglongbing in Florida. *Phytopathology* 98: 387–396 18944186 10.1094/PHYTO-98-4-0387

[RMerfa2022] Merfa MV, Naranjo E, Shantharaj D, De La Fuente L (2022) Growth of ‘*Candidatus* Liberibacter asiaticus’ in commercial grapefruit juice-based media formulations reveals common cell density-dependent transient behaviors. *Phytopathology* 112: 131–144 34340531 10.1094/PHYTO-06-21-0228-FI

[RMerfa2019] Merfa MV, Perez-Lopez E, Naranjo E, Jain M, Gabriel DW, De La Fuente L (2019) Progress and obstacles in culturing ‘*Candidatus* Liberibacter asiaticus’, the bacterium associated with huanglongbing. *Phytopathology* 109: 1092–1101 30998129 10.1094/PHYTO-02-19-0051-RVW

[RMishra2012] Mishra AR, Karimi D, Ehsani R, Lee WS (2012) Identification of citrus greening (HLB) using a vis-nir spectroscopy technique. *T Asabe* 55: 711–720

[RPark2018] Park JW, Louzada ES, Braswell WE, Stansly PA, da Graça JV, McCollum G, Rascoe JE, Kunta M (2018) A new diagnostic real-time PCR method for huanglongbing detection in citrus root tissue. *J Gen Plant Pathol* 84: 359–367

[RParker2014] Parker JK, Wisotsky SR, Johnson EG, Hijaz FM, Killiny N, Hilf ME, De La Fuente L (2014) Viability of ‘*Candidatus* Liberibacter asiaticus’ prolonged by addition of citrus juice to culture medium. *Phytopathology* 104: 15–26 23883155 10.1094/PHYTO-05-13-0119-R

[RRazi2012] Razi MFD, Khana IA, Jaskania MJ, Basrab SMA (2012) Graft transmission and biological indexing of huanglongbing on local citrus germplasm. *Sci Int (Lahore)* 24: 153–157

[RSchneider1968] Schneider H (1968) Anatomy of greening-diseased sweet orange shoots. *Phytopathology* 58: 1155–1160

[RSechler2009] Sechler A, Schuenzel EL, Cooke P, Donnua S, Thaveechai N, Postnikova E, Stone AL, Schneider WL, Damsteegt VD, Schaad NW (2009) Cultivation of ‘*Candidatus* Liberibacter asiaticus’, ‘*Ca*. L. africanus’, and ‘*Ca*. L. americanus’ associated with huanglongbing. *Phytopathology* 99: 480–486 19351243 10.1094/PHYTO-99-5-0480

[RSecor2009] Secor GA, Rivera VV, Abad JA, Lee IM, Clover GRG, Liefting LW, Li X, De Boer SH (2009) Association of ‘*Candidatus* Liberibacter solanacearum’ with zebra chip disease of potato established by graft and psyllid transmission, electron microscopy, and PCR. *Plant Dis* 93: 574–583 30764398 10.1094/PDIS-93-6-0574

[RStevenson2015] Stevenson HS, Wang Y, Muller R, Edelman DC (2015) Long-term stability of total RNA in RNAstable(R) as evaluated by expression microarray. *Biopreserv Biobank* 13: 114–122 25826008 10.1089/bio.2014.0068 PMC4442560

[RTakushi2007] Takushi T, Toyozato T, Kawano S, Taba S, Taba K, Ooshiro A, Numazawa M, Tokeshi M (2007) Scratch method for simple, rapid diagnosis of citrus huanglongbing using iodine to detect high accumulation of starch in the citrus leaves. *Ann Phytopathol Soc Jpn* 73: 3–8

[RTamura2011] Tamura K, Peterson D, Peterson N, Stecher G, Nei M, Kumar S (2011) MEGA5: Molecular evolutionary genetics analysis using maximum likelihood, evolutionary distance, and maximum parsimony methods. *Mol Biol Evol* 28: 2731–2739 21546353 10.1093/molbev/msr121 PMC3203626

[RTeixeira2005] Teixeira DDC, Saillard C, Eveillard S, Danet JL, Costa PI, Ayres AJ, Bové J (2005) ‘*Candidatus* Liberibacter americanus’, associated with citrus huanglongbing (greening disease) in São Paulo State, Brazil. *Int J Syst Evol Microbiol* 55: 1857–1862 16166678 10.1099/ijs.0.63677-0

[RThapa2021] Thapa N, Danyluk MD, Gerberich KM, Johnson EG, Dewdney MM (2021) Assessment of the effect of thermotherapy on ‘*Candidatus* Liberibacter asiaticus’ viability in woody tissue of citrus via graft-based assays and RNA assays. *Phytopathology* 111: 808–818 32976056 10.1094/PHYTO-04-20-0152-R

[RTrivedi2012] Trivedi P, He Z, Van Nostrand JD, Albrigo G, Zhou J, Wang N (2012) Huanglongbing alters the structure and functional diversity of microbial communities associated with citrus rhizosphere. *ISME J* 6: 363–383 21796220 10.1038/ismej.2011.100 PMC3260493

[Rvan2008] Van der Heijden MGA, Bardgett RD, van Straalen NM (2008) The unseen majority: Soil microbes as drivers of plant diversity and productivity in terrestrial ecosystems. *Ecol Lett* 11: 296–310 18047587 10.1111/j.1461-0248.2007.01139.x

[RWang2013] Wang N, Trivedi P (2013) Citrus huanglongbing: A newly relevant disease presents unprecedented challenges. *Phytopathology* 103: 652–665 23441969 10.1094/PHYTO-12-12-0331-RVW

[RWang2006] Wang Z, Yin Y, Hu H, Yuan Q, Peng G, Xia Y (2006) Development and application of molecular-based diagnosis for ‘*Candidatus* Liberibacter asiaticus’, the causal pathogen of citrus huanglongbing. *Plant Pathol* 55: 630–638

[RWulff2019] Wulff NA, Fassini CG, Marques VV, Martins EC, Coletti DAB, Teixeira DDC, Sanches MM, Bové JM (2019) Molecular characterization and detection of 16SrIII group phytoplasma associated with huanglongbing symptoms. *Phytopathology* 109: 366–374 30226423 10.1094/PHYTO-03-18-0081-R

[RZheng2018] Zheng Z, Chen J, Deng X (2018) Historical perspectives, management, and current research of citrus HLB in Guangdong Province of China, where the disease has been endemic for over a hundred years. *Phytopathology* 108: 1224–1236 30156499 10.1094/PHYTO-07-18-0255-IA

[RZheng2016] Zheng Z, Xu M, Bao M, Wu F, Chen J, Deng X (2016) Unusual five copies and dual forms of *nrdB* in “*Candidatus* Liberibacter asiaticus”: Biological implications and PCR detection application. *Sci Rep* 6: 39020 27958354 10.1038/srep39020 PMC5154197

