# Supplementary material for: Roots applicable, high sensitivity and specificity assay for the detection of Candidatus Liberibacter asiaticus in citrus roots and fruits
Source: Plant Biotechnol (Tokyo). 2024 Mar 25;41(1):27–34. doi: 10.5511/plantbiotechnology.23.1129a (PMC11500590; doi:10.5511/plantbiotechnology.23.1129a)
Supplement: Supplementary Data [file plantbiotechnology-41-1-23.1129a-s001.pdf]

Roots applicable, high sensitivity and specificity assay for the detection of *Candidatus* Liberibacter asiaticus in *citrus* roots and fruits

Zecheng Zhong[1], Yu Chen[2], Jinhua Liu[3], Wei Wang[3], Feng Zhou[3], Liu Hu[3], Jinlian Zhang[1,4], Tingsu Chen[4], Jiyu Xiang[1], Tingdong Li[1], Yingbin Wang[1,*], Shiyin Zhang[1,**], Shengxiang Ge[1,***], Jun Zhang[1], Ningshao Xia[1]

[1] National Institute of Diagnostics and Vaccine Development in Infectious Diseases, School of Public Health, Xiamen University, Xiamen 361102, China; [2] School of Medicine, Xiamen University, Xiamen, 361102, China; [3] Zhejiang Yang sheng tang Institute of Natural Medicine co., LTD, Hangzhou 310024, China; [4] Microbiology Research Institute, Guangxi Academy of Agricultural Sciences, Nanning, 530007, China

Zecheng Zhong and Yu Chen contribute equally to this paper.

[*]Yingbin Wang, ybwang@xmu.edu.cn Tel & Fax: 0592-2183111

[**]Shiyin Zhang, zhangshiyin@xmu.edu.cn Tel& Fax: 0592-2183111

[***]Shengxiang Ge, sxge@xmu.edu.cn Tel& Fax: 0592-2183111

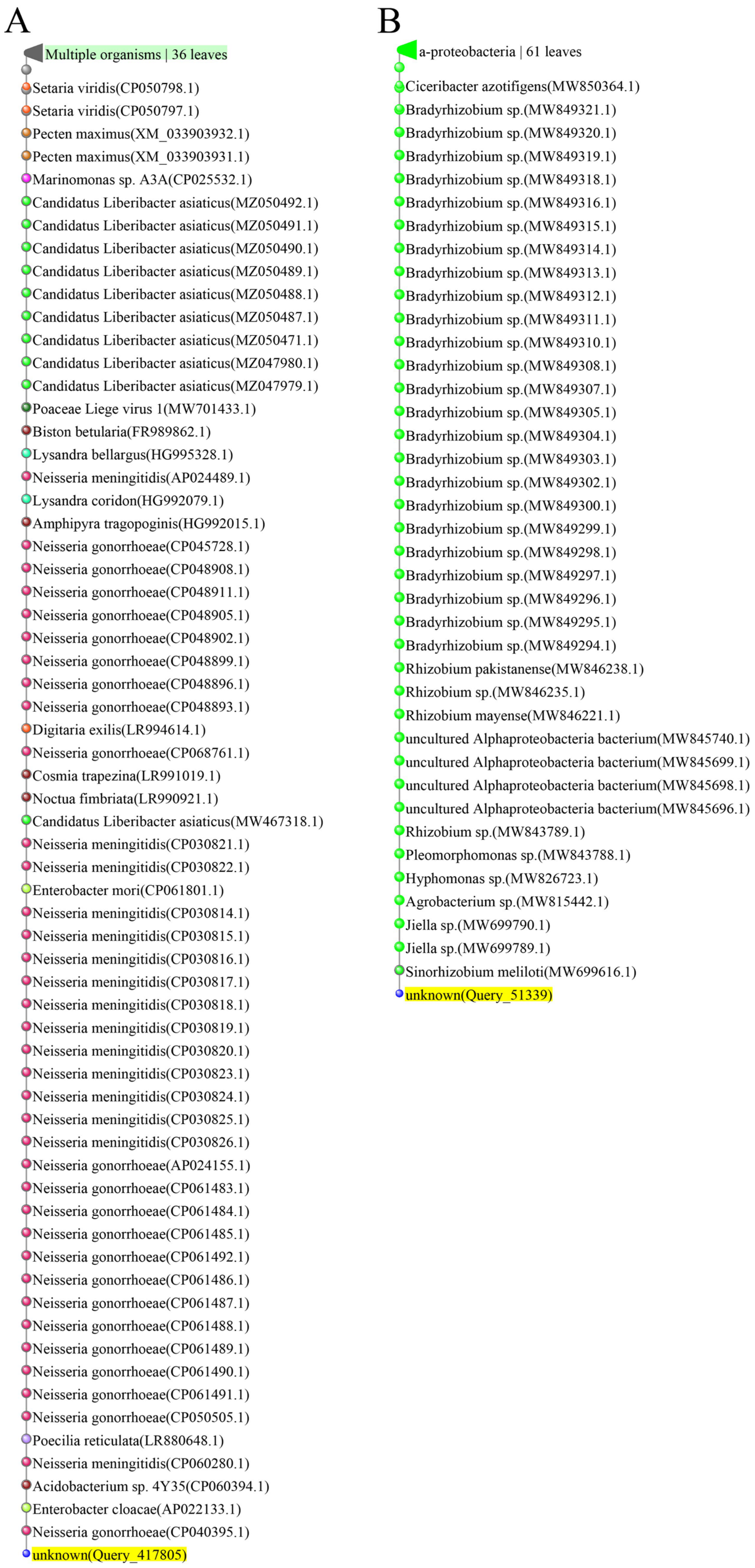
A
Multiple organisms | 36 leaves
Setaria viridis(CP050798.1)
Setaria viridis(CP050797.1)
Pecten maximus(XM_033903932.1)
Pecten maximus(XM_033903931.1)
Marinomonas sp. A3A(CP025532.1)
Candidatus Liberibacter asiaticus(MZ050492.1)
Candidatus Liberibacter asiaticus(MZ050491.1)
Candidatus Liberibacter asiaticus(MZ050490.1)
Candidatus Liberibacter asiaticus(MZ050489.1)
Candidatus Liberibacter asiaticus(MZ050488.1)
Candidatus Liberibacter asiaticus(MZ050487.1)
Candidatus Liberibacter asiaticus(MZ050471.1)
Candidatus Liberibacter asiaticus(MZ047980.1)
Candidatus Liberibacter asiaticus(MZ047979.1)
Poaceae Liege virus 1(MW701433.1)
Biston betularia(FR989862.1)
Lysandra bellargus(HG995328.1)
Neisseria meningitidis(AP024489.1)
Lysandra coridon(HG992079.1)
Amphipyra tragopoginis(HG992015.1)
Neisseria gonorrhoeae(CP045728.1)
Neisseria gonorrhoeae(CP048908.1)
Neisseria gonorrhoeae(CP048911.1)
Neisseria gonorrhoeae(CP048905.1)
Neisseria gonorrhoeae(CP048902.1)
Neisseria gonorrhoeae(CP048899.1)
Neisseria gonorrhoeae(CP048896.1)
Neisseria gonorrhoeae(CP048893.1)
Digitaria exilis(LR994614.1)
Neisseria gonorrhoeae(CP068761.1)
Cosmia trapezina(LR991019.1)
Noctua fimbriata(LR990921.1)
Candidatus Liberibacter asiaticus(MW467318.1)
Neisseria meningitidis(CP030821.1)
Neisseria meningitidis(CP030822.1)
Enterobacter mori(CP061801.1)
Neisseria meningitidis(CP030814.1)
Neisseria meningitidis(CP030815.1)
Neisseria meningitidis(CP030816.1)
Neisseria meningitidis(CP030817.1)
Neisseria meningitidis(CP030818.1)
Neisseria meningitidis(CP030819.1)
Neisseria meningitidis(CP030820.1)
Neisseria meningitidis(CP030823.1)
Neisseria meningitidis(CP030824.1)
Neisseria meningitidis(CP030825.1)
Neisseria meningitidis(CP030826.1)
Neisseria gonorrhoeae(AP024155.1)
Neisseria gonorrhoeae(CP061483.1)
Neisseria gonorrhoeae(CP061484.1)
Neisseria gonorrhoeae(CP061485.1)
Neisseria gonorrhoeae(CP061492.1)
Neisseria gonorrhoeae(CP061486.1)
Neisseria gonorrhoeae(CP061487.1)
Neisseria gonorrhoeae(CP061488.1)
Neisseria gonorrhoeae(CP061489.1)
Neisseria gonorrhoeae(CP061490.1)
Neisseria gonorrhoeae(CP061491.1)
Neisseria gonorrhoeae(CP050505.1)
Poecilia reticulata(LR880648.1)
Neisseria meningitidis(CP060280.1)
Acidobacterium sp. 4Y35(CP060394.1)
Enterobacter cloacae(AP022133.1)
Neisseria gonorrhoeae(CP040395.1)
unknown(Query_417805)
B
a-proteobacteria | 61 leaves
Ciceribacter azotifigens(MW850364.1)
Bradyrhizobium sp.(MW849321.1)
Bradyrhizobium sp.(MW849320.1)
Bradyrhizobium sp.(MW849319.1)
Bradyrhizobium sp.(MW849318.1)
Bradyrhizobium sp.(MW849316.1)
Bradyrhizobium sp.(MW849315.1)
Bradyrhizobium sp.(MW849314.1)
Bradyrhizobium sp.(MW849313.1)
Bradyrhizobium sp.(MW849312.1)
Bradyrhizobium sp.(MW849311.1)
Bradyrhizobium sp.(MW849310.1)
Bradyrhizobium sp.(MW849308.1)
Bradyrhizobium sp.(MW849307.1)
Bradyrhizobium sp.(MW849305.1)
Bradyrhizobium sp.(MW849304.1)
Bradyrhizobium sp.(MW849303.1)
Bradyrhizobium sp.(MW849302.1)
Bradyrhizobium sp.(MW849300.1)
Bradyrhizobium sp.(MW849299.1)
Bradyrhizobium sp.(MW849298.1)
Bradyrhizobium sp.(MW849297.1)
Bradyrhizobium sp.(MW849296.1)
Bradyrhizobium sp.(MW849295.1)
Bradyrhizobium sp.(MW849294.1)
Rhizobium pakistanense(MW846238.1)
Rhizobium sp.(MW846235.1)
Rhizobium mayense(MW846221.1)
uncultured Alphaproteobacteria bacterium(MW845740.1)
uncultured Alphaproteobacteria bacterium(MW845699.1)
uncultured Alphaproteobacteria bacterium(MW845698.1)
uncultured Alphaproteobacteria bacterium(MW845696.1)
Rhizobium sp.(MW843789.1)
Pleomorphomonas sp.(MW843788.1)
Hyphomonas sp.(MW826723.1)
Agrobacterium sp.(MW815442.1)
Jiella sp.(MW699790.1)
Jiella sp.(MW699789.1)
Sinorhizobium meliloti(MW699616.1)
unknown(Query_51339)

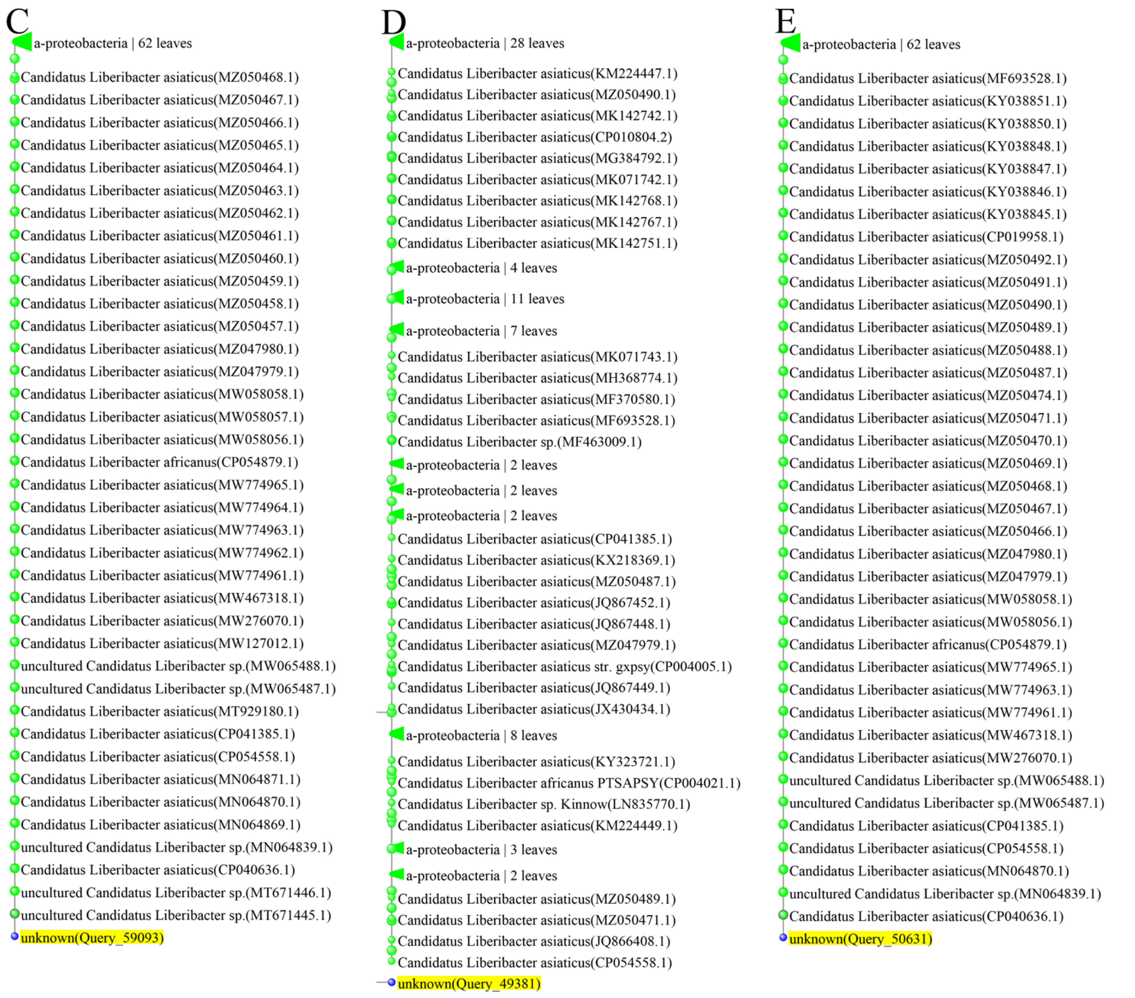

Supplementary Figure S1 BLASTn results of different primer set**. A** The BLASTn result of forward primer of (Li et al. 2006) showed in distance tree. **B** The BLASTn result of probe of (Li et al. 2006) showed in distance tree. **C** The BLASTn result of YSD-F showed in distance tree. **D** The BLASTn result of YSD-R showed in distance tree. **E** The BLASTn result of YSD-P showed in distance tree. The Query ID were yellow marked.

ID	Name	YSD-F	YSD-P	YSD-R
		CCAACGCAGGCTCATCTCTC	TCCAATAAAATCTTTCCCCCAATAGG	GTAGAAAAAGGTAGATTCCTACGC
NZ CP010804.1	**Candidatus Liberibacter asisticus A4**	••••••••••••••••••••	••••••••••••••••••••••••••	••••••••••••••••••••••••
CP001677.5	**Candidatus Liberibacter asisticus psy62**	••••••••••••••••••••	••••••••••••••••••••••••••	••••••••••••••••••••••••
NC 20549.1	**Candidatus Liberibacter asisticus gxpsy**	••••••••••••••••••••	••••••••••••••••••••••••••	••••••••••••••••••••••••
NZ AP014595.1	**Candidatus Liberibacter asisticus Ishi-1**	••••••••••••••••••••	••••••••••••••••••••••••••	••••••••••••••••••••••••
LIIM01000001.1	**Candidatus Liberibacter asisticus YCPsy**	••••••••••••••••••••	••••••••••••••••••••••••••	••••••••••••••••••••••••
DQ432003.1	**Candidatus Liberibacter asisticus Jiangxi-GC**	••••••••••••••••••••	••••••••••••••••••••••••••	•••••••••••••••C••••••••
L22533.1	**Candidatus Liberibacter africanus 16SrRNA gene**	••••••••••••••••••••	••••••••••••••••••••••••••	••••••••••••••••••••••••
CP004021.1	**Candidatus Liberibacter africanus PTSAPSY**	••••••••••••••••••••	••••••••••••••••••••••••••	••••••••••••••••••••••••
AOFG01000002.1	**Candidatus Liberibacter americanus PW_SP**	•T•••••G••T••••••T••	••••••••••••••••••T•T•A•••	•••••••••••••••••C•C••••
NC_022793.1	**Candidatus Liberibacter americanus str. Sao Paulo**	•T•••••G••T••••••T••	••••••••••••••••••T•T•A•••	•••••••••••••••••C•C••••
CP002371.1	**Candidatus Liberibacter solanacearum CLso-ZC1**	•T•••••G••••••••••••	•••••••••TCT••C•••TTCTC•••	•••••••••••••••••••C••••
GU373049.1	**Candidatus Liberibacter solanacearum 16SrRNA gene**	•T•••••G••••••••••••	•••••••••TCT••C•••TTCTC•••	•••••••••••••••••••C••••
NR_102476.2	**Liberibacter crescens BT-1**	TGGCG•••••••T•A•A•AT	G•A•G•CG•GCGCG•ATTTAT•CGA•	AC••G•T••C•C•••AA•G•GT••
U45329.1	**Agrobacterium vitis**	TGGCG•••••••T•A•A•AT	G•A•G•CG•GCGCG••G•AAG•CGA•	ACG•••T•GCTCC••AA•TGGAAT
JN175336.1	**Carbophilus carboxidus CIP 105722**	TGGCG•••••••T•A•A•AT	G•A•G•CG•GCGCGC•G•AAGGCGA•	ACG•••T••CTC•••AA•T•GT••
AJ011762.1	**Chelatobacter heintzii DSM6450T**	TGGCG•••••••T•A•A•AT	G•A•G•CG•GCGCGC•G•AAGGCGA•	ACG•••T••CTC•••AA•T•GT••
U29386.1	**Rhizobium leguminosarum bv. Viciae**	TGGCG•••••••T•A•A•AT	G•A•G•CG•GCGCGC•G•AAGGCGA•	ACG•••T••C•C•••AA•T•GT••
EF173322.1	**Sinorhizobium sp. MKI8**	TGGCG•••••••T•A•A•AT	G•A•G•CG•GCGCG•AT•AAT•CGA•	ACG•••T••CCC•••AA•T•GGA•
U28916.1	**Rhizobium etli CFN 42**	TGGCG•••••••T•A•A•AT	G•A•G•CG•GCGCGC•G•AAGGCGA•	ACG•••T••C•C•••AA•T•GT••
AM921640.1	**Ensifer adhaerens**	TGGCG•••••••T•A•A•AT	G•A•G•CG•GCGCGC•G•AAGGCGA•	ACG•••T••C•C•••AA•G•GT••
EF592609.1	**Kaistia soli strain 5YN9-8**	TGGCG••••••CT•A•A•AT	G•A•G•CG••CGCGC•G•AAGGCGA•	ACG•••C••CCA•••AA•T•TG••
MF966236.1	**Bradyrhizobium sp. Strain N195**	•ATTG••G••A•TTCGC•GT	CTACG•TTCTCT••CTT•G•GGAC•A	ACG•G•T•CGT•GGG•T•T•GTAG
GU553033.1	**Uncultured bacterium clone M-20 16S ribosomal RNA gene, partial sequence**	••••••••••••••••••••	••••••••••••••••••••••••••	••••••••••••••••••••••••
XM_022952774.1	**Stylophora pistillata uncharacterized LOC111345489**	••••T•••••TGT•A•A••T	TGAGGGCT•TATCT•C•G•CTC•TTG	AC••C•••C•A•TC•••TT•GAAG
KT768936.1	**Uncultured bacterium clone GCSTWT_16S_429 16S ribosomal RNA gene**	A•••••••••••••••••••	AGC•GG••ACCGC•AGGCC•TGC•CT	AATCT•TG•••A••••••••••••
KR267580.1	**Uncultured bacterium clone 1170 16S ribosomal RNA gene**	A•••••••••••••••••••	AGGTGCCTA•TTT•CTAGG•G•C•CT	CC•CT••••••C••••••••••••
XM_038921115.1	**Brettanomyces nanus uncharacterized protein**	T•••••••••••••••••••	••••TA•T•TCTGCCTT•ATCGATCC	A••••TG••AT•GACA••T•TTA•
CP064812.1	**Brettanomyces nanus strain CBS 1945 chromosome 1**	T•••••••••••••••••••	••••TA•T•TCTGCCTT•ATCGATCC	A••••TG••AT•GACA••T•TTA•
MT603686.1	**Uncultured bacterium clone T2-f12-09 16S ribosomal RNA gene, partial sequence**	••••••••••••••••••AT	AG•GCG•GGCC•GAAGGT••CCCGCT	CC•CT•CG••••••••••••••••
MN788571.1	**Uncultured bacterium clone BL-C11 16S ribosomal RNA gene, partial sequence**	••••••••••••••••••AT	AG•GCG•GGCC•GAAGGT••CCCGCT	C••AGTTGTCCCCC•C•TTG•G•T
MN788565.1	**Uncultured bacterium clone BL-B05 16S ribosomal RNA gene, partial sequence**	••••••••••••••••••AT	AG•GCG•GGCC•GAAGGT••CCCGCT	C••AGTTGTCCCCC•C•TTG•G•T
MN788555.1	**Uncultured bacterium clone BH2-D12 16S ribosomal RNA gene, partial sequence**	••••••••••••••••••AT	AG•GCG•GGCC•GAAGGT••CCCGCT	C••AGTTGTCCCCC•C•TTG•G•T
MG489510.1	**Uncultured bacterium clone OTU_4101 16S ribosomal RNA gene, partial sequence**	••••••••••••••••••AT	AG•••CTGCC•AAGCAG••TTTCCTC	•••TT•GC•C•AGTT•C••CGT•T
LC209328.1	**Uncultured gamma proteobacterium gene for 16S ribosomal RNA, partial sequence, clone: SJC-46**	••••••••••••••••••AT	AGTG•••GCT•A•AAATAGAGGCCAT	•••TT•GCCC•AGTT•C••CGG•T
MF950988.1	**Uncultured bacterium clone B09- G1511061598-H38- _M13F(-47)_SA.ab1 16S ribosomal RNA gene, partial sequence**	••••••••••••••••••AT	AG•GCG•GGCC•GAAGGT••CCCGCT	CC•CT•CG•••C••••••••••••
MF950986.1	**Uncultured bacterium clone G08- G1511061400-H10- _M13F(-47)_SA.ab1 16S ribosomal RNA gene, partial sequence**	••••••••••••••••••AT	AG•GCG•GGCC•GAAGGT••CCCGCT	CC•CT•CG•••C••••••••••••
KY579181.1	**Uncultured bacterium clone OTU_3811 16S ribosomal RNA gene, partial sequence**	••••••••••••••••••AA	AG•GCC•GGCC•GAAGGT••CCAGCT	CA•CTTT•••AC••••••••••••
KY577934.1	**Uncultured bacterium clone OTU_1328 16S ribosomal RNA gene, partial sequence**	••••••••••••••••••AA	GA•GCCTGG•C•CGAAGG•TCCC•A•	A••CCTT••••C••••••••••••
LT169800.1	**Uncultured bacterium partial 16S rRNA gene, clone 1911_48_2003**	••••••••••••••••••AT	AG•GCA•GGCC•GAAGGT••CC•GCT	CCGCT•T•••••A•••••••••••
LT169345.1	**Uncultured bacterium partial 16S rRNA gene, clone 1611_21_172**	••••••••••••••••••AT	AGTGCA•GGCC•GAAGGT••CC•GCT	CCGCT•TG•••C••••••••••••
KT858322.1	**Uncultured bacterium clone Gammaproteobacteria_100_OTU7858 16S ribosomal RNA gene, partial sequence**	••••••••••••••••••AT	AGTGT••GGCC•GAAGGT••CCA•CT	CCGCT•CG••••••••••••••••
KR268671.1	**Uncultured Francisellaceae bacterium clone 2152 16S ribosomal RNA gene, partial sequence**	••••••••••••••••••AT	GG•G•C•GC•C••AAGTG•••CCTTT	CC•CC•T••••C••••••••••••
JX441412.1	**Uncultured bacterium clone YD1600-1 16S ribosomal RNA gene, partial sequence**	••••••••••••••••••AT	AG•GCA•GGCC•GAAGGT••CC•GCT	CCGCT•T••••C•A••••••••••
LN807575.1	**Uncultured proteobacterium partial 16S rRNA gene, clone AB240522**	••••••••••••••••••AT	AG•GCG•GGCC•GAAGGT••CCCGCT	CC•CTTTG••••••••••••••••
LN800514.1	**Uncultured proteobacterium partial 16S rRNA gene, clone denovo7700**	••••••••••••••••••AT	AG•GCG•GGTC•AAAGAT••CCCGCT	CC•CT•TG•••C••••••••••••
NR_044848.2	**Methylomicrobium pelagicum strain ACM 3505 16S ribosomal RNA, partial sequence**	••••••••••••••••••AT	AG•GCG•GGTC•GAAGGT••CCCGCT	CC•CT•CC•••C••••••••••••
XM_038407170.2	**PREDICTED: Dermochelys coriacea transmembrane protein 86A (TMEM86A), transcript variant X3, mRNA**	AT•••AT••••C•••A•••T	G•TCCC•CTGATG•AT•ATAGGAGCA	•A••C•GGCCC••••CC••CT•TT
KF799891.1	**Uncultured bacterium clone SanDiego_a7361 16S ribosomal RNA gene, partial sequence**	••••••••••••••••••AT	AG•GCA•GGCC•GAAGGT••CC•GCT	CC•CT•TG•••C••••••••••••
HG970993.1	**Uncultured bacterium partial 16S rRNA gene, clone 17**	••••••••••••••••••AT	GG•G•C•GC•C••AAGTG•••CCTTT	CC•CC•T••••C••••••••••••
JX170276.1	**Uncultured bacterium clone AJ-U-CDˇ52 16S ribosomal RNA gene, partial sequence**	••••••••••••••••••AT	GG•G•C•GC•C••AAGTG•••CCTTT	CC•CC•T••••C••••••••••••
JX170258.1	**Uncultured bacterium clone AJ-U-CDˇ169 16S ribosomal RNA gene, partial sequence**	••••••••••••••••••AT	GG•G•C•GC•C••AAGTG•••CCTTT	CC•CC•T••••C••••••••••••
KC606264.1	**Uncultured bacterium clone CarbonSeq024_022210_A12 16S ribosomal RNA gene, partial sequence**	••••••••••••••••••AT	AG•G•A•GGCC•GAAGGT••CC•CCT	CC•CT•TG•••C••••••••••••
KC668927.1	**Uncultured bacterium clone 12B04 16S ribosomal RNA gene, partial sequence**	••••••••••••••••••AT	AGTGCGTGGCC•GAAGGT••C•CGCT	CCGCT•TG•••C••••••••••••
JX100373.1	**Uncultured bacterium clone HLUCs353 16S ribosomal RNA gene, partial sequence**	••••••••••••••••••AT	AGTGT••GGCC•GAAGGT••CCA•CT	CC•CT•CG•••C••••••••••••
KC922843.1	**Uncultured bacterium clone 300cmOC0174 16S ribosomal RNA gene, partial sequence**	••••••••••••••••••AT	AG•GCG•GGTC•GAAGAT••CCCGCT	CC•CT•TG•••C••••••••••••
KC922783.1	**Uncultured bacterium clone 300cmOC0105 16S ribosomal RNA gene, partial sequence**	••••••••••••••••••AT	AG•GCG•GGTC•GAAGAT••CCCGCT	CC•CT•TG•••C••••••••••••
KC922760.1	**Uncultured bacterium clone 300cmOC0078 16S ribosomal RNA gene, partial sequence**	••••••••••••••••••AT	AG•GCG•GGTC•GAAGAT••CCCGCT	CC•CT•TG•••C••••••••••••
KC922727.1	**Uncultured bacterium clone 300cmOC0043 16S ribosomal RNA gene, partial sequence**	••••••••••••••••••AT	AG•GCG•GGTC•GAAGAT••CCCGCT	••••GGCGTATGC•G•ATTAG••T
KC922699.1	**Uncultured bacterium clone 300cmOC0007 16S ribosomal RNA gene, partial sequence**	••••••••••••••••••AT	AG•GCG•GGTC•GAAGAT••CCCGCT	CC•CT•TG•••C••••••••••••
KC922641.1	**Uncultured bacterium clone 0cmOC0227 16S ribosomal RNA gene, partial sequence**	••••••••••••••••••AT	AG•GCG•GGTC•GAAGAT••CCCGCT	CCCCCGT•G••CGT••G•GGTATT
KC922498.1	**Uncultured bacterium clone 0cmOC0053 16S ribosomal RNA gene, partial sequence**	••••••••••••••••••AT	AG•GCG•GGTCT•ACGAT••CCCGCT	CC•CT•TG•••C••••••••••••
KC574820.1	**Uncultured bacterium clone lagoon3_C14 16S ribosomal RNA gene, partial sequence**	••••••••••••••••••AT	AG•GCA•GGCC•GAAGGT••CC•GCT	CCGCT•T•••••A•••••••••••
KC841538.1	**Uncultured microorganism clone YLˇRII09 16S ribosomal RNA gene, partial sequence**	••••••••••••••••••AA	A••G•••••TCT••••T•T•TC•GCC	CAGTTTG•GCCAGAT•A••C••••
MH764455.2	**Methylomicrobium sp. strain RS1 16S ribosomal RNA gene, partial sequence**	••••••••••••••••••AT	AG•GCG•GGCC•GAAGGT••CCCGCT	CC•CT•CG•••C••••••••••••
JN914793.1	**Uncultured microorganism clone GF13U7304JVIUD 16S ribosomal RNA gene, partial sequence**	••••••••••••••••••AT	AGTGCA•GGCC•GAAGGT••CC•GCT	CCGCT•TG•••C••••••••••••
JN914464.1	**Uncultured microorganism clone GF13U7304I5YZ7 16S ribosomal RNA gene, partial sequence**	••••••••••••••••••AT	AGTGCA•GGCC•GAAGGT••CCCGCT	CGCCT•TG•••C••••••••••••
JN914463.1	**Uncultured microorganism clone GF13U7304IMTZD 16S ribosomal RNA gene, partial sequence**	••••••••••••••••••AT	AGTGCA•GGCC•GAAGGT••CC•C•C	CGCCT•TG•••C••••••••••••
JN914459.1	**Uncultured microorganism clone GF13U7304JMRR1 16S ribosomal RNA gene, partial sequence**	••••••••••••••••••AT	AGTGCA•GGCC•GAAAGGT•CCCT•C	CCGCT•TG•••C••••••••••••
JN914458.1	**Uncultured microorganism clone GF13U7304J0VYY 16S ribosomal RNA gene, partial sequence**	••••••••••••••••••AT	AGTGCA•GGCC•GAAAGGT•CCCT•C	CCGCT•TG•••C••••••••••••
JN914456.1	**Uncultured microorganism clone GF13U7304JDM0Y 16S ribosomal RNA gene, partial sequence**	••••••••••••••••••AT	AGTGCA•GGCC•GAAGGT••CC•GCT	CCGCT•TG•••C••••••••••••
JN914455.1	**Uncultured microorganism clone GF13U7304IFEWZ 16S ribosomal RNA gene, partial sequence**	••••••••••••••••••AT	AGTGCA•GGCC•GAAGGT••CC•GCT	CCGCT•TG•••C••••••••••••
JN914454.1	**Uncultured microorganism clone GF13U7304JS45H 16S ribosomal RNA gene, partial sequence**	••••••••••••••••••AT	AGTGCA•GGCC•GAAGGT••CC•GCT	CCGCT•TG•••C••••••••••••
JN914453.1	**Uncultured microorganism clone GF13U7304JRQZX 16S ribosomal RNA gene, partial sequence**	••••••••••••••••••AT	AGTGCA•GGCC•GAAGGT••CC•GCT	CCGCT•TG•••C••••••••••••
JN914452.1	**Uncultured microorganism clone GF13U7304IQCJW 16S ribosomal RNA gene, partial sequence**	••••••••••••••••••AT	AGTGCA•GGCC•GAAGGT••CC•GCT	CCGCT•TG•••C••••••••••••
JN914451.1	**Uncultured microorganism clone GF13U7304IWWH4 16S ribosomal RNA gene, partial sequence**	••••••••••••••••••AT	AGTGCA•GGCC•GAAGGT••CC•GCT	CCGCT•TG•••C••••••••••••
MT914441.1	**Uncultured Legionellales bacterium clone ZOTU 4344 16S ribosomal RNA gene, partial sequence**	••••••••••••••••••AT	AG•G•A•GCT•A•AAATAGAGGCTTC	CC•CT•CG•••C••••••••••••
GU553033.1	**Uncultured bacterium clone Mˇ20 16S ribosomal RNA gene, partial sequence**	••••••••••••••••••••	••••••••••••••••••••••••••	••••••••••••••••••••••••
XM_033214887.1	**PREDICTED: Trachypithecus francoisi solute carrier family 9 member B1 (SLC9B1), mRNA**	G••CTAA•••TATTCTAA•G	AGTTG•••••••••••••••••••AT	A•CAC•••GA•••T•ACT•C•TTT
XM_023220118.3	**PREDICTED: Piliocolobus tephrosceles solute carrier family 9 member B1 (SLC9B1), mRNA**	G••CTAA•••TATTCTAA•G	AGTTG•••••••••••••••••••AT	A•CAC•••GA•••T•ACT•C•TTT
XM_031664395.1	**PREDICTED: Papio anubis solute carrier family 9 member B1 (SLC9B1), transcript variant X11, mRNA**	G••CTAA•••TATTCTAA•G	AGTTG•••••••••••••••••••AT	A•CAC•••GA•••T•ACT•C•TTT
XR_004182743.1	**PREDICTED: Papio anubis solute carrier family 9 member B1 (SLC9B1), transcript variant X10, misc_RNA**	G••CTAA•••TATTCTAA•G	AGTTG•••••••••••••••••••AT	A•CAC•••GA•••T•ACT•C•TTT
XM_031664394.1	**PREDICTED: Papio anubis solute carrier family 9 member B1 (SLC9B1), transcript variant X9, mRNA**	G••CTAA•••TATTCTAA•G	AGTTG•••••••••••••••••••AT	A•CAC•••GA•••T•ACT•C•TTT
XM_031664393.1	**PREDICTED: Papio anubis solute carrier family 9 member B1 (SLC9B1), transcript variant X8, mRNA**	G••CTAA•••TATTCTAA•G	AGTTG•••••••••••••••••••AT	A•CAC•••GA•••T•ACT•C•TTT
XM_017958903.2	**PREDICTED: Papio anubis solute carrier family 9 member B1 (SLC9B1), transcript variant X7, mRNA**	G••CTAA•••TATTCTAA•G	AGTTG•••••••••••••••••••AT	A•CAC•••GA•••T•ACT•C•TTT
XM_031664392.1	**PREDICTED: Papio anubis solute carrier family 9 member B1 (SLC9B1), transcript variant X6, mRNA**	G••CTAA•••TATTCTAA•G	AGTTG•••••••••••••••••••AT	A•CAC•••GA•••T•ACT•C•TTT
XM_031664391.1	**PREDICTED: Papio anubis solute carrier family 9 member B1 (SLC9B1), transcript variant X5, mRNA**	G••CTAA•••TATTCTAA•G	AGTTG•••••••••••••••••••AT	A•CAC•••GA•••T•ACT•C•TTT
XM_031664390.1	**PREDICTED: Papio anubis solute carrier family 9 member B1 (SLC9B1), transcript variant X4, mRNA**	G••CTAA•••TATTCTAA•G	AGTTG•••••••••••••••••••AT	A•CAC•••GA•••T•ACT•C•TTT
XM_031664389.1	**PREDICTED: Papio anubis solute carrier family 9 member B1 (SLC9B1), transcript variant X3, mRNA**	G••CTAA•••TATTCTAA•G	AGTTG•••••••••••••••••••AT	A•CAC•••GA•••T•ACT•C•TTT
XM_031664388.1	**PREDICTED: Papio anubis solute carrier family 9 member B1 (SLC9B1), transcript variant X2, mRNA**	G••CTAA•••TATTCTAA•G	AGTTG•••••••••••••••••••AT	A•CAC•••GA•••T•ACT•C•TTT
XM_031664387.1	**PREDICTED: Papio anubis solute carrier family 9 member B1 (SLC9B1), transcript variant X1, mRNA**	G••CTAA•••TATTCTAA•G	AGTTG•••••••••••••••••••AT	A•CAC•••GA•••T•ACT•C•TTT
XM_010363329.2	**PREDICTED: Rhinopithecus roxellana solute carrier family 9 member B1 (SLC9B1), mRNA**	G••CTAA•••TATTCTAA•G	AGTTG•••••••••••••••••••AT	A•CAC•••GA•••T•ACT•C•TTT
XM_001110263.4	**PREDICTED: Macaca mulatta solute carrier family 9 member B1 (SLC9B1), transcript variant X8, mRNA**	G••CTAA•••TATTCTAA•G	AGTTG•••••••••••••••••••AT	A•CAC•••GA•••T•ACT•C•TTT
XM_015138889.2	**PREDICTED: Macaca mulatta solute carrier family 9 member B1 (SLC9B1), transcript variant X7, mRNA**	G••CTAA•••TATTCTAA•G	AGTTG•••••••••••••••••••AT	A•CAC•••GA•••T•ACT•C•TTT
XM_015138888.2	**PREDICTED: Macaca mulatta solute carrier family 9 member B1 (SLC9B1), transcript variant X6, mRNA**	G••CTAA•••TATTCTAA•G	AGTTG•••••••••••••••••••AT	A•CAC•••GA•••T•ACT•C•TTT
XM_001110224.4	**PREDICTED: Macaca mulatta solute carrier family 9 member B1 (SLC9B1), transcript variant X5, mRNA**	G••CTAA•••TATTCTAA•G	AGTTG•••••••••••••••••••AT	A•CAC•••GA•••T•ACT•C•TTT
XM_028848651.1	**PREDICTED: Macaca mulatta solute carrier family 9 member B1 (SLC9B1), transcript variant X4, mRNA**	G••CTAA•••TATTCTAA•G	AGTTG•••••••••••••••••••AT	A•CAC•••GA•••T•ACT•C•TTT
XM_015138886.2	**PREDICTED: Macaca mulatta solute carrier family 9 member B1 (SLC9B1), transcript variant X3, mRNA**	G••CTAA•••TATTCTAA•G	AGTTG•••••••••••••••••••AT	A•CAC•••GA•••T•ACT•C•TTT
XM_028848650.1	**PREDICTED: Macaca mulatta solute carrier family 9 member B1 (SLC9B1), transcript variant X2, mRNA**	G••CTAA•••TATTCTAA•G	AGTTG•••••••••••••••••••AT	A•CAC•••GA•••T•ACT•C•TTT
XM_015138885.2	**PREDICTED: Macaca mulatta solute carrier family 9 member B1 (SLC9B1), transcript variant X1, mRNA**	G••CTAA•••TATTCTAA•G	AGTTG•••••••••••••••••••AT	A•CAC•••GA•••T•ACT•C•TTT
XM_027644659.1	**PREDICTED: Corapipo altera gammaˇglutamylamine cyclotransferase (GGACT), transcript variant X2, mRNA**	GTTTG•ACATT•TTATAT•G	•AT•TA•••••••••••••••••••A	T•GA•CTG•A••G••AC•TA•A•T
XM_027644650.1	**PREDICTED: Corapipo altera gammaˇglutamylamine cyclotransferase (GGACT), transcript variant X1, mRNA**	GTTTG•ACATT•TTATAT•G	•AT•TA•••••••••••••••••••A	T•GA•CTG•A••G••AC•TA•A•T
XM_027674682.1	**PREDICTED: Neopelma chrysocephalum gammaˇglutamylamine cyclotransferase (GGACT), transcript variant X3, mRNA**	•TTTG•ATATT•TTATAT•G	•AT•TA•••••••••••••••••••A	••GA•CTG•A••G••AC•TA•A•T
XM_027674681.1	**PREDICTED: Neopelma chrysocephalum gammaˇglutamylamine cyclotransferase (GGACT), transcript variant X2, mRNA**	•TTTG•ATATT•TTATAT•G	•AT•TA•••••••••••••••••••A	••GA•CTG•A••G••AC•TA•A•T
XM_027674680.1	**PREDICTED: Neopelma chrysocephalum gammaˇglutamylamine cyclotransferase (GGACT), transcript variant X1, mRNA**	•TTTG•ATATT•TTATAT•G	•AT•TA•••••••••••••••••••A	••GA•CTG•A••G••AC•TA•A•T
XM_025384949.1	**PREDICTED: Theropithecus gelada solute carrier family 9 member B1 (SLC9B1), transcript variant X2, mRNA**	G••CTAA•••TATTCTAA•G	AGTTG•••••••••••••••••••AT	A•CAC•••GA•••T•ACT•C•TTT
XM_025384948.1	**PREDICTED: Theropithecus gelada solute carrier family 9 member B1 (SLC9B1), transcript variant X1, mRNA**	G••CTAA•••TATTCTAA•G	AGTTG•••••••••••••••••••AT	A•CAC•••GA•••T•ACT•C•TTT
XM_011749184.2	**PREDICTED: Macaca nemestrina solute carrier family 9 member B1 (SLC9B1), transcript variant X7, mRNA**	G••CTAA•••TATTCTAA•G	AGTTG•••••••••••••••••••AT	A•CAC•••GA•••T•ACT•C•TTT
XM_011749185.2	**PREDICTED: Macaca nemestrina solute carrier family 9 member B1 (SLC9B1), transcript variant X6, mRNA**	G••CTAA•••TATTCTAA•G	AGTTG•••••••••••••••••••AT	A•CAC•••GA•••T•ACT•C•TTT
XM_011749183.2	**PREDICTED: Macaca nemestrina solute carrier family 9 member B1 (SLC9B1), transcript variant X5, mRNA**	G••CTAA•••TATTCTAA•G	AGTTG•••••••••••••••••••AT	A•CAC•••GA•••T•ACT•C•TTT
XM_011749182.2	**PREDICTED: Macaca nemestrina solute carrier family 9 member B1 (SLC9B1), transcript variant X4, mRNA**	G••CTAA•••TATTCTAA•G	AGTTG•••••••••••••••••••AT	A•CAC•••GA•••T•ACT•C•TTT
XM_011749181.2	**PREDICTED: Macaca nemestrina solute carrier family 9 member B1 (SLC9B1), transcript variant X3, mRNA**	G••CTAA•••TATTCTAA•G	AGTTG•••••••••••••••••••AT	A•CAC•••GA•••T•ACT•C•TTT
XM_011749180.2	**PREDICTED: Macaca nemestrina solute carrier family 9 member B1 (SLC9B1), transcript variant X2, mRNA**	G••CTAA•••TATTCTAA•G	AGTTG•••••••••••••••••••AT	A•CAC•••GA•••T•ACT•C•TTT
XM_011749179.2	**PREDICTED: Macaca nemestrina solute carrier family 9 member B1 (SLC9B1), transcript variant X1, mRNA**	G••CTAA•••TATTCTAA•G	AGTTG•••••••••••••••••••AT	A•CAC•••GA•••T•ACT•C•TTT
XM_024527892.1	**PREDICTED: Physcomitrella patens uncharacterized LOC112286214 (LOC112286214), transcript variant X5, mRNA**	GA•GAATTTAGCGC•TGGAA	GAA•T•••••••••••••••••••AT	•CT•••••TCTCTAGACAAAC•CA
XM_024527889.1	**PREDICTED: Physcomitrella patens uncharacterized LOC112286214 (LOC112286214), transcript variant X2, mRNA**	GA•GAATTTAGCGC•TGGAA	GAA•T•••••••••••••••••••AT	•CT•••••TCTCTAGACAAAC•CA
MG576283.1	**Myroides odoratimimus strain RS RED 16S ribosomal RNA gene, partial sequence**	TTG•TA•GCATC•CATG••A	GGTCGAT•••••••••••••••••••	••CA••••••CA•ATC•T••G•TA
XM_019411973.1	**PREDICTED: Nicotiana attenuata pyruvate dehydrogenase E1 component subunit alpha-3, chloroplastic-like (LOC109244821**	GA••AAAG•AGCTG•••T•A	CT•••A•GGG•A•••TTAT••G•G•A	••••C•G••AA•GAGG•AGCCATG
XM_018071177.1	**PREDICTED: Manacus vitellinus gammaˇglutamylamine cyclotransferase (GGACT), mRNA**	GTTTGAATATT•TTATAT•G	•AT•TA•••••••••••••••••••A	••GA•CTG•A••G••AC•TA•A•T
XM_017877079.1	**PREDICTED: Rhinopithecus bieti solute carrier family 9 member B1 (SLC9B1), mRNA**	G••CTAA•••TATTCTAA•G	AGTTG•••••••••••••••••••AT	A•CAC•••GA•••T•ACT•C•TTT
XM_017816627.1	**PREDICTED: Lepidothrix coronata gammaˇglutamylamine cyclotransferase (GGACT), transcript variant X2, mRNA**	GTTTGAATATT•TTATAT•G	•AT•TA•••••••••••••••••••A	•C•••GT•••AATA••C•T••AAT
XM_017816626.1	**PREDICTED: Lepidothrix coronata gammaˇglutamylamine cyclotransferase (GGACT), transcript variant X1, mRNA**	GTTTGAATATT•TTATAT•G	•AT•TA•••••••••••••••••••A	•C•••GT•••AATA••C•T••AAT
KU830850.1	**Uncultured bacterium clone PS1B_2535 16S ribosomal RNA gene, partial sequence**	••••••G•••C••••C•AG	GG•••••••••••••••••••TCCCC	CC•CCCC•G••C••G•G••C••••
XM_005555564.2	**PREDICTED: Macaca fascicularis solute carrier family 9, subfamily B (NHA1, cation proton antiporter 1), member 1 (SLC9B**	G••CTAA•••TATTCTAA•G	AGTTG•••••••••••••••••••AT	A•CAC•••GA•••T•ACT•C•TTT
XM_027730776.2	**PREDICTED: Pipra filicauda gammaˇglutamylamine cyclotransferase (GGACT), transcript variant X2, mRNA**	GTTTGAATATT•TTATAT•G	•AT•TA•••••••••••••••••••A	••GA•CTG•A••G••AC•TA•A•T
XM_039389415.1	**PREDICTED: Pipra filicauda gammaˇglutamylamine cyclotransferase (GGACT), transcript variant X1, mRNA**	G••CTAA•••TATTCTAA•G	AGTTG•••••••••••••••••••AT	A•CAC•••GA•••T•ACT•C•TTT
XM_007999416.2	**PREDICTED: Chlorocebus sabaeus solute carrier family 9 member B1 (SLC9B1), mRNA**	G••CTAA•••TATTCTAA•G	AGTTG•••••••••••••••••••AT	A•CAC•••GA•••T•ACT•C•TTT
GU553033.1	**Uncultured bacterium clone Mˇ20 16S ribosomal RNA gene, partial sequence**	••••••••••••••••••••	••••••••••••••••••••••••••	CC•C••••••••••••••••••••
JN038715.1	**Uncultured gamma proteobacterium clone MP-R42 16S ribosomal RNA gene, partial sequence**	••C•GTGT••••GGC•GTC•	G•TTGGT•GGC•A••A•••••TC•	CAC•••••••••••••••••••••
LC276020.1	**Uncultured bacterium gene for 16S ribosomal RNA, partial sequence, clone: JZK60_16S**	AG•C•AGCTA•C•G••GTCG	G•TTGGTG•GC•A••A••T••CC•AC	CC•C••••••••••••••••••••
KX270140.1	**Uncultured gamma proteobacterium clone 6AP4 16S ribosomal RNA gene, partial sequence**	AG•C•AGCTA•C•G••GTCG	G•TTGGTG•GC•A••A••T••CC•AC	CC•C••••••••••••••••••••
KU830931.1	**Uncultured bacterium clone PS1B_3410 16S ribosomal RNA gene, partial sequence**	••C•GTGT••••G•••GTC•	G••TTGGT•GC•A••A•••••CC•	CACT••••••••••••••••••••
KU991830.1	**Ensifer sp. strain Lotˇara1 16S ribosomal RNA gene, partial sequence**	•••••••G••••••••CT•T	•••GGAT•••••••••••••G•AG••	•••••••G••••••••••••••••
KR188916.1	**Uncultured bacterium clone HF40 16S ribosomal RNA gene, partial sequence**	AG•C•AGCTA•C•G••GTCG	G•TTGGTG•GC•A••A••T••CC•AC	CC•C••••••••••••••••••••
KT776139.1	**Uncultured bacterium clone GCSTWT_16S_8061 16S ribosomal RNA gene, partial sequence**	AG•C•AGCTA•GG••••TCG	C•TTGGTG•GC••••A••T••CC•AC	CC•C••••••••••••••••••••
KP050720.1	**Uncultured bacterium clone BA_53 16S ribosomal RNA gene, partial sequence**	AG•C•AGCTA•C•G••GTCG	G•TTGGTG•GC•A••A••T••CC•AC	CC•C••••••••••••••••••••
KF521158.1	**Uncultured bacterium clone 4442 16S ribosomal RNA gene, partial sequence**	•T•••••G••••••••••••	C•A•TA••TC•T•CC••TT•TCAG••	•••••••••••••••••••C••••
JX106652.1	**Uncultured bacterium clone MID29_22008 16S ribosomal RNA gene, partial sequence**	•••••••G••••••••CT•T	•••CGAT•••••••••T•••G•AG•A	•••••••G••••••••••••••••
HE660430.1	**Uncultured bacterium partial 16S rRNA gene, clone 12258**	•••••••G••••••••••••	CT•C•AT•••••••••••A••AG••	•••••••••••••••••••C••••
JN651990.1	**Uncultured bacterium clone BSL34B-11 16S ribosomal RNA gene, partial sequence**	AG•C•AGCTA•C•G••GTCG	G•TTGGT•GGC•A••A•••••CC•AC	CC•C••••••••••••••••••••
AB597551.1	**Uncultured gamma proteobacterium gene for 16S rRNA, partial sequence, clone: G01**	AG•C•AGCTA•C•G••GTCG	G•TTGGT•GGC•A••A•••••CC•AC	CC•C••••••••••••••••••••
JF403559.1	**Uncultured soil bacterium clone GO0VNXF07H4ZB0 16S ribosomal RNA gene, partial sequence**	AG•C•AGCTA•C•G••GTCG	G•TTGGTG•GC•A••A••T••CC•AC	CC•C••••••••••••••••••••
JF395944.1	**Uncultured soil bacterium clone GO0VNXF07IAU51 16S ribosomal RNA gene, partial sequence**	AG•C•AGCTA•C•G••GTCG	G•TTGGTG•GC•A••A••T••CC•AC	CC•C••••••••••••••••••••
JF390944.1	**Uncultured soil bacterium clone GO0VNXF07IA75Y 16S ribosomal RNA gene, partial sequence**	AG•C•AGCTA•C•G••GTCG	C•TTGGTG•GC•A••A••T••CC•AC	CC•C••••••••••••••••••••
JF388130.1	**Uncultured soil bacterium clone GO0VNXF07H7IZ4 16S ribosomal RNA gene, partial sequence**	AG•C•AGCTA•C•G••GTCG	G•TTGGTG•GC•A••A••T••CC•AC	CC•C••••••••••••••••••••
JF387998.1	**Uncultured soil bacterium clone GO0VNXF07H1GDP 16S ribosomal RNA gene, partial sequence**	AG•C•AGCTA•C•G••GTCG	G•TTGGTG•GC•A••A••T••CC•AC	CC•C••••••••••••••••••••
JF387808.1	**Uncultured soil bacterium clone GO0VNXF07IMMSE 16S ribosomal RNA gene, partial sequence**	AG•C•AGCTA•C•G••GTCG	G•TTGGTG•GC•A••A••T••CC•AC	CC•C••••••••••••••••••••
JF387797.1	**Uncultured soil bacterium clone GO0VNXF07ILIG8 16S ribosomal RNA gene, partial sequence**	•AG••CAGCTAC•G••GTCG	G•TTGGTG•GC•A••A••T••CC•AC	CC•C••••••••••••••••••••
JF387491.1	**Uncultured soil bacterium clone GO0VNXF07IM5CD 16S ribosomal RNA gene, partial sequence**	AG•C•AGCTA•C•G••GTCG	G•TTGGTG•GC•A••A••T••CC•AC	CC•C••••••••••••••••••••
JF387275.1	**Uncultured soil bacterium clone GO0VNXF07IFUJ6 16S ribosomal RNA gene, partial sequence**	AG•C•AGCTA•C•G••GTCG	G•TTGGTG•GC•A••A••T••CC•AC	CC•C••••••••••••••••••••
JF386692.1	**Uncultured soil bacterium clone GO0VNXF07IJANE 16S ribosomal RNA gene, partial sequence**	AG•C•AGCTA•C•G••GTCG	G•TTGGTG•GC•A••A••T••CC•AC	CC•C••••••••••••••••••••
JF378131.1	**Uncultured soil bacterium clone GO0VNXF07H2SD5 16S ribosomal RNA gene, partial sequence**	•AG••CAGCTAC•G••GTCG	G•TTGGTG•GC•A••A••T••CC•AC	CC•C••••••••••••••••••••
JF303548.1	**Uncultured bacterium clone ACB01C09 16S ribosomal RNA gene, partial sequence**	AG•C•AGCTA•C•G••GTCG	G•TTGGTG•GC•A••A••T••CC•AC	••••••••••••••••••••••••
GU795798.1	**Uncultured bacterium clone Sang_727 16S ribosomal RNA gene, partial sequence**	•••••••G••••••••CT•T	•••CGAT•••••••••••••G•AG••	•••••••••••••••••••C••••

Supplementary Figure S2 Alignments of oligonucleotide binding regions**.** The panels show six available sequences of *C*Las, aligned to *Ca*. L. africanus, *Ca*. L. americanus, *Ca*. L. solanacearum and the other closely related strains and other unrelated species genes. Dots represent identical nucleotides compared with the *CLas* A4 sequence. Nucleotide substitutions are specified. Red arrows: primers and probes designed areas.

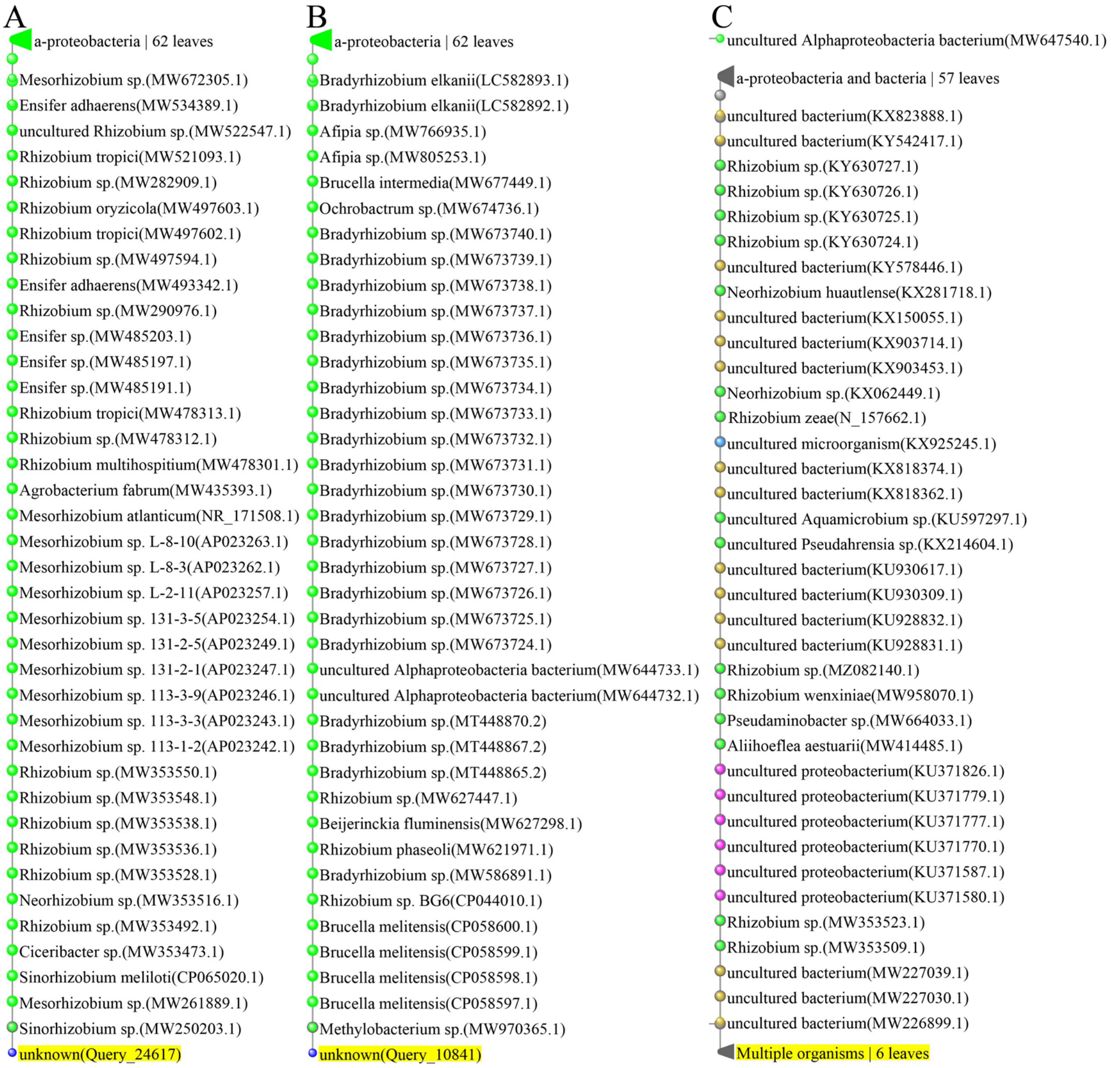

Supplementary Figure S3 The BLAST alignment results of amplicon sequencing data**.** Non-specific PCR amplicons generated by HLBasfP assay were then gel purified, cloned into the TA cloning system and sequenced. The nucleotide sequence of the 5 amplicons were listed in Table S4. BLAST alignment results showed that no matches with CLas. **A** Sample 1, collected from root. **B** Sample 2, collected from *Cogongrass*. **C** Sample 3, 4, and 5, collected from root, leaf, and *Polygonum L*, respectively.

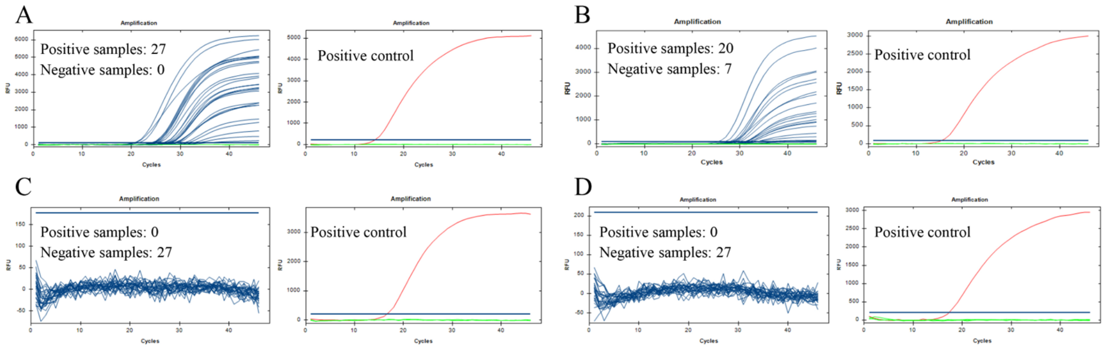

Supplementary Figure S4 The fluorescence amplification curve of four CLas assays for healthy plant roots detection**. A** The roots of four different healthy plant species collected from five different places and detected by four CLas assays. Co-detection assay with HLBasfP. **B** Single DNA detection assay with HLBasfP. **C** Co-detection assay with YSD-FRP. **D** Single DNA detection assay with YSD-FRP. Blue curve: sample; Red curve: positive control; Green curve: negative control.

Supplementary Table S1. General information of PCR primers in this study.

Name	Type	Sequence (5′ → 3′)	Amplicon size (bp)	Gene	PCR format	Reference
YSD-F	Forward	CCAACGCAGGCTCATCTCTC	109 bp	16S rRNA gene	Taqman	This study
YSD-R	Reverse	GCGTAGGRATCTACCTTTTTCTAC				
YSD-P	Probe	FAM-TCCAATAAAATCTTTCCCCCAATAGG-BHQ1				
HLBas	Forward	TCGAGCGCGTATGCAATACG	76 bp	16S rRNA gene	Taqman	(Li et al. 2006)
HLBr	Reverse	GCGTTATCCCGTAGAAAAAGGTAG				
HLB-P	Probe	FAM-AGACGGGTGAGTAACGCG-BHQ1				
RNRf	Forward	CATGCTCCATGAAGCTACCC	80 bp	nrdB, β-subunit of ribonucleotide reductase	Taqman	(Zheng et al. 2016)
RNRr	Reverse	GGAGCATTTAACCCCACGAA				
RNRp	Probe	FAM-CCTCGAAATCGCCTATGCAC-BHQ1				

Supplementary Table S2. The results of the specificity verification of co-detection assays.

Family	Plant type	Tssues	Healthy/Diseased	Cq (YSD-FRP)	Cq (HLBasfP)
Rutaceae	*Navel orange*	leaves	diseased	23.16 (1)	22.34 (1)
			healthy	N/A (10)	38.3 (1), N/A (9)
		roots	diseased	27.26 (1)	26.14 (1)
			healthy	N/A (10)	32.23 (1), 28.20 (1), N/A (8)
	Citrus medica L	leaves, roots	healthy	N/A (1)	N/A (1)
Non-Rutaceae	*Peperomia puteolata, Schefflera, Laurel, Peperomia tetraphylla, Camphor, Delonix regia, Indigofera, Mangifera indica L, Cogongrass, Bauhinia L, Lactuca sativa L, Oldenlandia corymbosa L, Ficus microcarpa L. f, Polygonum L, Bidens pilosa L, Bougainvillea spectabilis Willd, Lantana camara L, Hibiscus syriacus L, Lagerstroemia indica L, Cinnamomum camphora (L.) Presl, Cyperus rotundus L, Loropetalum chinense (R. Br.) Oliver, Aglaia odorata Lour, Duranta repens L, Codiaeum variegatum (L.) A. Juss, Gardenia jasminoides Ellis, Taxus wallichiana var. chinensis (Pilg.) Florin, Carmona microphylla (Lam.) G. Don, Oxalis corniculata L, Photinia serrulata Lindl, Podocarpus macrophyllus (Thunb.) D. Don, Nerium indicum Mill, Herba Paederiae, Plumeria rubra L. cv. Acutifolia, Euphorbia hirta, Catharanthus roseus (L.) G. Don, Zinnia elegans Jacq, Hymenocallis littoralis (Jacq.) Scalisb, Calliandra haematocephala Hassk*	leaves	healthy	N/A (40)	33.50 (1 of *Polygonum L*), 31.85 (1 of *Cogongrass*), N/A (38)
	Indigofera, Mangifera indica , Cogongrass, Bauhinia L Lactuca sativa L, Oldenlandia corymbosa L, Ficus microcarpa L. f, Polygonum L, Bidens pilosa L, Bougainvillea spectabilis Willd, Lantana camara L, Hibiscus syriacus L, Lagerstroemia indica L, Cinnamomum camphora (L.) Presl, Cyperus rotundus L	roots	healthy	N/A (15)	N/A (15)

Numbers were underlined represent the number of samples (each sample was collected from a separate tree). N/A = not detected.

Supplementary Table S3. The Cq value of four CLas assays for healthy plant roots detection.

Assay	Plant species and source area	Sample 1	Sample 2	Sample 3
Co-detection assay with HLBasfP	**Navel orange** - *Xiamen*	29.41	31.79	27.01
	**Navel orange** - *Ganzhou*	32.26	31.84	28.86
	**Orah mandarin** - *Guilin*	27.43	31.30	23.20
	**Navel orange** - *Guilin*	22.23	25.88	26.86
	**Navel orange** - *Wuming, Nanning*	30.94	29.20	29.55
	**Orah mandarin** - *Guangxi Academy of Agricultural Sciences*	25.85	22.18	28.01
	**Navel orange** - *Guangxi Academy of Agricultural Sciences*	25.98	25.71	29.26
	**Lemon tree** - *Guangxi Academy of Agricultural Sciences*	29.41	26.21	29.28
	**Citrus poonensis** - *Guangxi Academy of Agricultural Sciences*	28.04	28.31	27.67
Single DNA detection assay with HLBasfP	**Navel orange** - *Xiamen*	31.66	N/A	29.91
	**Navel orange** - *Ganzhou*	N/A	N/A	N/A
	**Orah mandarin** - *Guilin*	32.42	N/A	28.22
	**Navel orange** - *Guilin*	26.87	31.69	34.22
	**Navel orange** - *Wuming, Nanning*	N/A	N/A	31.87
	**Orah mandarin** - *Guangxi Academy of Agricultural Sciences*	28.93	29.42	30.44
	**Navel orange** - *Guangxi Academy of Agricultural Sciences*	29.36	29.41	31.14
	**Lemon tree** - *Guangxi Academy of Agricultural Sciences*	31.09	29.90	30.97
	**Citrus poonensis** - *Guangxi Academy of Agricultural Sciences*	30.94	31.54	30.53
Co-detection assay with YSD-FRP	**Navel orange** - *Xiamen*	N/A	N/A	N/A
	**Navel orange** - *Ganzhou*	N/A	N/A	N/A
	**Orah mandarin** - *Guilin*	N/A	N/A	N/A
	**Navel orange** - *Guilin*	N/A	N/A	N/A
	**Navel orange** - *Wuming, Nanning*	N/A	N/A	N/A
	**Orah mandarin** - *Guangxi Academy of Agricultural Sciences*	N/A	N/A	N/A
	**Navel orange** - *Guangxi Academy of Agricultural Sciences*	N/A	N/A	N/A
	**Lemon tree** - *Guangxi Academy of Agricultural Sciences*	N/A	N/A	N/A
	**Citrus poonensis** - *Guangxi Academy of Agricultural Sciences*	N/A	N/A	N/A
Single DNA detection assay with YSD-FRP	**Navel orange** - *Xiamen*	N/A	N/A	N/A
	**Navel orange** - *Ganzhou*	N/A	N/A	N/A
	**Orah mandarin** - *Guilin*	N/A	N/A	N/A
	**Navel orange** - *Guilin*	N/A	N/A	N/A
	**Navel orange** - *Wuming, Nanning*	N/A	N/A	N/A
	**Orah mandarin** - *Guangxi Academy of Agricultural Sciences*	N/A	N/A	N/A
	**Navel orange** - *Guangxi Academy of Agricultural Sciences*	N/A	N/A	N/A
	**Lemon tree** - *Guangxi Academy of Agricultural Sciences*	N/A	N/A	N/A
	**Citrus poonensis** - *Guangxi Academy of Agricultural Sciences*	N/A	N/A	N/A

N/A = not detected.

Supplementary Table S4. The nucleotide sequence of the 5 amplicons generated by HLBasfP assay.

Type	Sequence (5′ → 3′)
Sample 1	AGCGGCAGACGGGTGAGTAACGCGTGGGAAT
Sample 2	AGCGGCAGACGGGTGAGTAACGCGTGGGAAC
Sample 3	AGCGGCAGACGGGTGAGTAACACGTGGGAAT
Sample 4	AGCGGCAGACGGGTGAGTAACACGTGGGAAT
Sample 5	AGCGGCAGACGGGTGAGTAACACGTGGGAAT

References

Li W, Hartung JS, Levy L (2006). Quantitative real-time PCR for detection and identification of *Candidatus* Liberibacter species associated with citrus huanglongbing. *J Microbiol Methods* 66: 104-115

Zheng Z, Xu M, Bao M, Wu F, Chen J, Deng X (2016). Unusual Five Copies and Dual Forms of nrdB in "*Candidatus* Liberibacter asiaticus": Biological Implications and PCR Detection Application. *Sci Rep* 6: 39020
